# Modelling how plant cell-cycle progression leads to cell size regulation

**DOI:** 10.1371/journal.pcbi.1011503

**Published:** 2023-10-20

**Authors:** Daniel Williamson, William Tasker-Brown, James A. H. Murray, Angharad R. Jones, Leah R. Band

**Affiliations:** 1 Centre for Mathematical Medicine and Biology, School of Mathematical Sciences, University of Nottingham, Nottingham, United Kingdom; 2 Cardiff School of Biosciences, Cardiff University, Sir Martin Evans Building, Museum Avenue, Cardiff, United Kingdom; 3 Division of Plant and Crop Sciences, School of Biosciences, University of Nottingham, Sutton Bonington Campus, Loughborough, United Kingdom; IFOM - the Firc Insitute of Molecular Oncology, ITALY

## Abstract

Populations of cells typically maintain a consistent size, despite cell division rarely being precisely symmetrical. Therefore, cells must possess a mechanism of “size control”, whereby the cell volume at birth affects cell-cycle progression. While size control mechanisms have been elucidated in a number of other organisms, it is not yet clear how this mechanism functions in plants.

Here, we present a mathematical model of the key interactions in the plant cell cycle. Model simulations reveal that the network of interactions exhibits limit-cycle solutions, with biological switches underpinning both the G1/S and G2/M cell-cycle transitions. Embedding this network model within growing cells, we test hypotheses as to how cell-cycle progression can depend on cell size. We investigate two different mechanisms at both the G1/S and G2/M transitions: (i) differential expression of cell-cycle activator and inhibitor proteins (with synthesis of inhibitor proteins being independent of cell size), and (ii) equal inheritance of inhibitor proteins after cell division. The model demonstrates that both these mechanisms can lead to larger daughter cells progressing through the cell cycle more rapidly, and can thus contribute to cell-size control. To test how these features enable size homeostasis over multiple generations, we then simulated these mechanisms in a cell-population model with multiple rounds of cell division. These simulations suggested that integration of size-control mechanisms at both G1/S and G2/M provides long-term cell-size homeostasis.

We concluded that while both size independence and equal inheritance of inhibitor proteins can reduce variations in cell size across individual cell-cycle phases, combining size-control mechanisms at both G1/S and G2/M is essential to maintain size homeostasis over multiple generations. Thus, our study reveals how features of the cell-cycle network enable cell-cycle progression to depend on cell size, and provides a mechanistic understanding of how plant cell populations maintain consistent size over generations.

## Author summary

Within growing biological tissues, cells maintain approximately constant sizes during multiple rounds of growth and division. This is essential for functioning, as biochemical processes would break down if cells became very large or very small. Studies have suggested that cell size is controlled via the dynamics of key proteins within the cell cycle, causing larger cells to divide sooner than smaller cells. However, cell-size control in plants is an open question. Here, we investigated plant cell-size control by creating a mathematical model of the plant cell cycle within a cell population, incorporating the interactions between the cell-cycle proteins within each cell. We demonstrated that our cell-cycle model exhibits cyclic behaviour with one-way switches at the two key cell-cycle transition points. We investigated two alternative mechanisms for cell size control: size-independent synthesis of cell-cycle regulators and equal inheritance of specific cell-cycle proteins. We showed that both mechanisms can reduce variations in cell size. We found that combining two mechanisms acting at both cell-cycle transitions is essential to maintain cell sizes over multiple rounds of cell division. Thus, our study demonstrates how features of the cell-cycle network enable plant cell populations to maintain constant sizes and thus form viable plant tissues.

## 1 Introduction

Plants contain mitotically active cells [1] which frequently undergo non-symmetric division, introducing cell size variability [2, 3]. Continued asymmetric division over generations could, in principle, lead to a scenario in which some cell lineages become successively larger over generations, while other lineages become vanishingly small. This would have substantial disadvantages, as excessively large cells may be limited by the rate at which molecules are able to diffuse across them, while small cells may fail to assemble intracellular structures [4]. It is evidently necessary for organisms to possess a homeostatic mechanism regulating cell size at division but the molecular basis for this mechanism in plants is not yet fully understood.

Cell division is regulated by the cell cycle, which can be divided into four main stages: (Gap 1) G1-phase, (Synthesis) S-phase, (Gap 2) G2-phase, and (mitosis) M-phase. During both gap phases, the cell grows and produces new macromolecules (although the length and therefore relative extent of growth in the two phases may be different). In S-phase, the cell replicates its DNA; finally, at M-phase, the cell divides into two daughter cells, distributing the DNA equally between the daughters (referred to as cytokinesis). The transitions from G1 to S (G1/S) and from G2 to M (G2/M) are thus key cell-cycle checkpoints, which are controlled by a network of regulatory proteins and are influenced by many environmental inputs [5].

When cell size homeostasis is active, larger cells generally undergo division sooner after birth than smaller cells. Thus larger cells generally add less volume per cell cycle and, in this way, cell size asymmetries are evened out over generations [4, 6, 7]. This type of size control was observed experimentally in yeasts and algae over 40 years ago [8–12] and has, more recently, been observed in plants [2, 3, 13, 14].

Many models have considered how cells maintain a constant distribution of sizes [6, 7, 15–18]. These models typically suggest that cells use protein concentrations as an internal scale to measure their size and trigger cell-cycle transitions. For example, suppose a certain protein acts as an inhibitor of the G1/S transition. As long as the inhibitor concentration is high, G1/S will be blocked and the cell will remain in G1-phase. However, should the inhibitor concentration reduce, for example due to growth-induced dilution, then the cell would undergo the G1/S transition.

Most proteins are synthesised in proportion to cell size and therefore cannot be used as an internal scale to measure growth [19–22]. These proteins, which are synthesised at a rate which is proportional to cell size, are referred to as size-dependent. Some size-independent exceptions (which are synthesised at a fixed rate, regardless of cell size) have been identified, and, together with their interplay with size-dependent proteins, have been suggested to provide a mechanism for cell-size control [20]. In budding yeast, for example, the concentration of the G1-inhibitor Whi5 reduces throughout the G1 phase [20]. Such dilution can lead to size control, with larger cells undergoing the G1/S transition sooner, if the Whi5 synthesis per cell cycle is independent of cell size (leading to the observed lower Whi5 concentration at birth in larger cells) [20, 23]. There is evidence that the correlation between Whi5 concentration and birth size is due to Whi5 synthesis occurring primarily during S/G2/M at a rate largely independent of cell size [20]; however, this suggestion was later questioned [23] and other models can be envisaged, for example, involving rapid turnover. This inhibitor-dilution mechanism with size-independent Whi5 has been explored in a theoretical model [15], which also simulated how size-independence may be created via saturation leading to protein synthesis depending on gene copy number rather than cell size [15, 24]. Similar size-independent inhibitor-dilution mechanisms have been suggested in fission yeast [25].

In addition to the concept of size control being achieved by the presence of size-dependent proteins, other mechanisms have also been considered. A recent study by D’Ario *et al.* proposed a novel mechanisms for size control at the G1/S transition in plants in which DNA is used as an internal scale [14]. Initially the G1/S transition is blocked by the cell-cycle inhibitor, KRP. KRP is diluted by cell volume growth until a threshold concentration is reached, at which time the G1/S transition occurs, and the cell cycle proceeds. KRP then associates strongly with chromatin, and free KRP molecules are rapidly degraded until almost all of the remaining KRP molecules within the cell are bound to chromatin. At the point of cytokinesis, DNA is distributed equally between the two daughter cells. Since the remaining KRP is predominantly bound with the chromatin, the concentration of KRP is lower in the larger daughter, so that the threshold KRP concentration is reached more quickly. In this way, larger cells have a shorter cell cycle and thus add less volume before division, establishing size control.

Many cell-cycle regulators are highly conserved across eukaryotes despite separation by billions of years of evolution [26], significantly including the cyclin dependent kinases (CDK) which are activators of cell-cycle progression [27]. In addition, functionally similar transcription factors which promote the expression of genes required for DNA synthesis have been identified in different eukaryotes (E2F in animals and plants, SBF in yeast). Likewise, E2F/SBF inhibitors have been found to have highly analogous functions (RB in animals and its functional equivalents, RBR in plants and Whi5 in yeast) [26].

While cell-cycle networks in different eukaryotes show similar features, cell size control mechanisms may differ. Although empirical studies have revealed cell size control in a wide variety of single- and multicellular systems, the observed dynamics (as characterised by the relationships between cell size at birth, cell-cycle length and cell size at division) vary from population to population. Empirically, most systems have been shown to exhibit “sloppy cell size control” as described in mammalian cell culture [28] and budding yeast [18] where the probability of cell division increases with cell size. Phenomenologically this means that observed behaviours, including those of plant cells [3], lie somewhere between the predicted behaviours of a population of perfect “adders”, where division is triggered once the cell has added a fixed volume, and perfect “sizers”, where division is triggered once a cell has reached a critical volume [17], with some more closely matching adders (e.g. human primary cells [29]) and others more closely resembling sizers (e.g. fission yeast [30] and mouse epidermal cells [31]). Thus, insights into size control in one organism cannot necessarily be generalised to other organisms.

Understanding control of the cell cycle is of particular relevance in plants where most development is post-embryonic and growth is both indeterminate and highly plastic to the environment. Since plant cells are immobile and surrounded by a semi-rigid wall they maintain relationships with neighbouring cells, and the timing of divisions and the size of cells becomes relevant in the construction of tissues from composing cells. The independent origin of multicellularity in plants means that many links to controllers of morphogenesis and development are distinct. Likely as a consequence of these considerations, the plant cell cycle has important differences from that of animals or yeasts/fungi [32–34]. In yeast a single CDK controls both transitions, and there appears to be two thresholds of kinase activity required for first the G1/S transition, and then a higher level for G2/M [35]. Higher eukaryotes in contrast exert additional control by using different CDKs at the two transitions. There are two major classes of CDK involved in direct cell cycle regulation in plants. Plant CDKA is highly homologous to yeast CDC28/cdc2 and mammalian CDK1 [36]. Notably the major cyclin-dependent kinase operating at the G2/M transition in plants is in a different subclass to all other known CDKs and is unique in showing strongly cell-cycle-regulated transcription, a behavior more normally associated with cyclins. The relevance of plant cell cycle and cell-size control to growth and development together with the unique aspects of its regulation mean that a conceptual modeling framework to understand these differences is of importance.

Several previous studies have developed plant-specific mathematical models of the cell cycle and cell-size control. For example, Zhao *et al.* [37] published a model of the G1/S transition in plants which exhibited resettable bistability (this being a model of the G1/S transition in isolation rather than a complete cell-cycle model). A very comprehensive model of the plant cell cycle was published by Ortiz-Gutiérrez *et al.* [38] which involves a large number of proteins and interactions. The authors demonstrated using Boolean analysis that their model admits stable limit-cycle solutions. A further cell-cycle model was presented in [39], albeit focusing on how hormones control the transition to endoreduplication (when the cell division ceases). A primarily empirical study by Jones *et al.* [13] includes a simple cell-cycle model driven by CDK activity. The authors assume that cytokinesis is initiated once a threshold of CDK activity is reached, suggesting that CDK activity being size-dependent would enable cell-size control.

Some questions remain, however. First, it has been shown experimentally that plants exhibit size control at both the G1/S and G2/M transitions [13]. Size control at the G2/M transition in plants is, to our knowledge, unexplained. Secondly, published models of plant-cell-size control rely on the existence of phenomenological concentration thresholds to explain size control [13] and are not explained as an emergent property of the protein interaction network.

In this article, we propose a plant cell-cycle model, which we use to investigate how size control can exist as an emergent property of a network of interacting cell-cycle regulators. The proteins controlling the plant cell cycle are well-attested (see [40–43] for reviews). However, cell-cycle regulation is highly complicated; more than 70 core cell-cycle proteins have so far been identified [44]. Our aim is to propose a parsimonious model which meets the following criteria:

1. The model must admit limit-cycle solutions
2. The model must exhibit hysteresis at the major transitions, G1/S and G2/M [5].
3. The transitions must be driven by the accumulation of activated cyclin-dependent kinases [35].

Hysteresis is a key feature of the classic Novák-Tyson model of the cell cycle [5]. Although the concentrations of activator and inhibitor proteins (e.g. CDK and KRP in plants) vary continuously over time, the transition from G1-phase to S-phase is a discrete switch in cell state (likewise for G2/M). Hysteretic behaviour can explain how this discrete switch occurs. In essence, G1-phase corresponds to a stable state of the protein network. As the activator protein accumulates and the inhibitor is diluted, an additional stable state (S-phase) is created. Further accumulation and dilution eventually eliminate the G1 state, forcing the cell to switch spontaneously to the S state. A hysteretic system is also robust to fluctuations in activator and inhibitor concentration. If the G1/S transition were a straightforward threshold mechanism (the system is in G1 below a key threshold, and in S above the threshold) then stochastic variation in activator concentration would cause the cell to vacillate between G1 and S. Hysteresis ensures that the cell cycle is irreversible, but ultimately resettable.

Having demonstrated that our proposed parsimonious cell-cycle network model exhibits limit cycle solutions (§3.1), with hysteresis at both the G1/S and G2/M transitions (§3.2 and §3.3), we embed this network model within a growing cell to test hypotheses as to how cell-cycle progression can depend on cell size. We demonstrate two different mechanisms for cell-size control at both G1/S and G2/M transitions. For G1/S (§3.4), we show that the transition depends on cell size if either KRP is equally inherited (as proposed in [14]) or key inhibitor proteins are size independent (as modelled in budding yeast in [15]). While activated cyclin-dependent kinases are established activator proteins, the model reveals that either KRP or RBR can take the role of size-independent inhibitor proteins, enabling size control at the G1/S transition. Similar mechanisms are shown to enable cell size control at the G2/M transition (§3.5). The model predicts that size control of the G2/M transition can be created either through size independence or equal inheritance of the CDK-inhibitor protein SMR. To investigate whether these mechanisms lead to cell-size homeostasis over multiple generations, we integrate the network model into a cell population model and simulate multiple rounds of cell division (§3.6 and §3.7). These simulations suggest that combining size-control mechanisms at both the G1/S and G2/M transitions leads to robust size control. With these size-control mechanisms, the model predicts how key genetic mutations affect cell size and cell-cycle phase duration, consistent with previous experimental observations. Thus, our modelling reveals size control to be an emergent property of the network structure and protein dynamics, and provides a mechanistic explanation of how plant cell populations achieve size homeostasis.

## 2 Model description

### 2.1 A parsimonious plant cell-cycle network model

**Fig 1:**
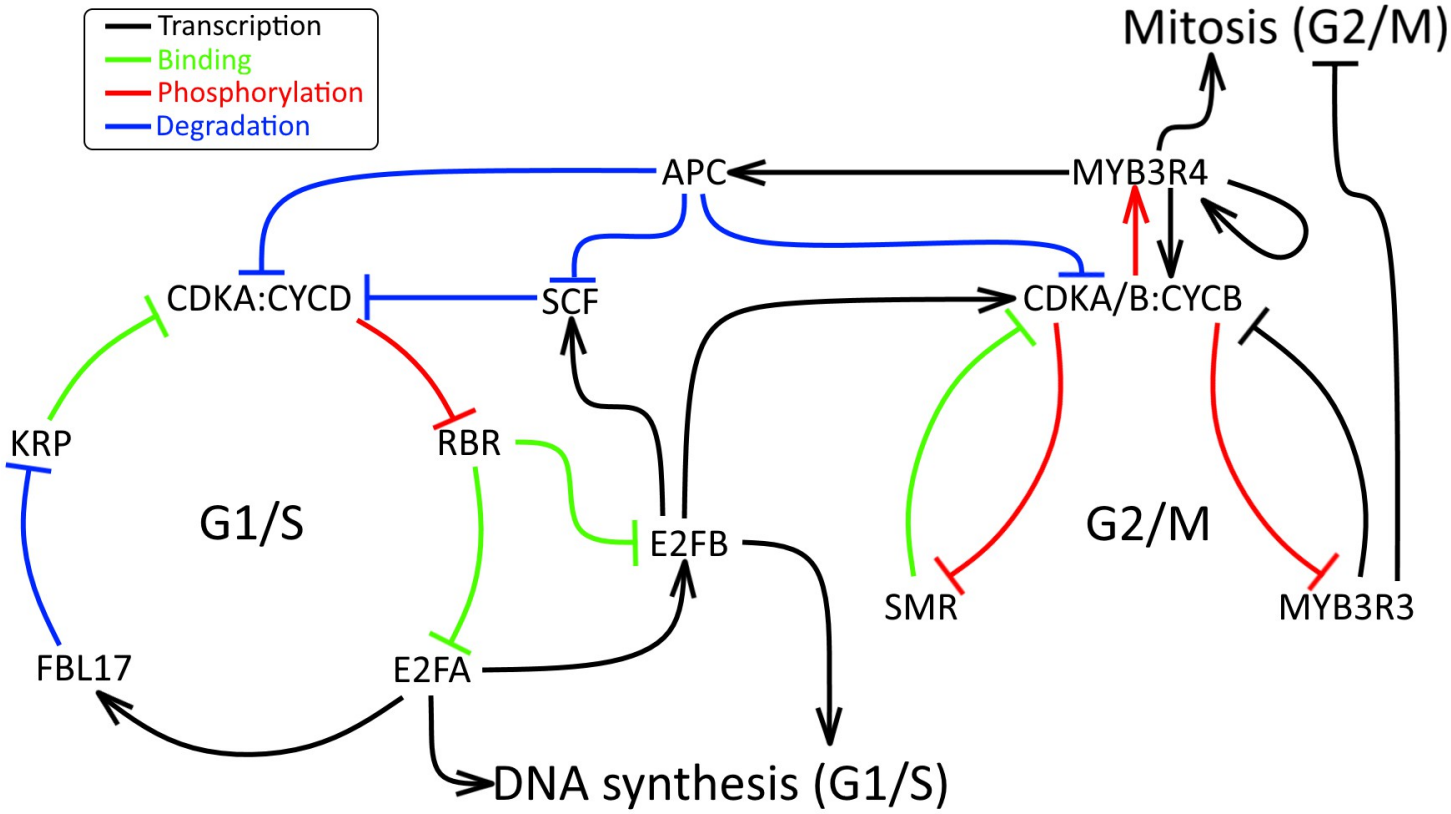
Schematic representation of the cell-cycle protein regulatory network.

Below we enumerate the set of proteins and protein interactions which have been included in the cell-cycle network model. This information is represented schematically in Fig 1.

#### G1-S components

- **CDKA:CYCD** (complex of cyclin dependent kinase A with cyclin D). CDKA is a highly conserved kinase [34] which, in association with cyclins, promotes progression through the cell cycle [45]. CDK-cyclin activity increase at both the G1/S and G2/M transitions leading to the phosphorylation of numbers of target proteins resulting in the onset of DNA synthesis and mitosis, respectively [34, 46]. CDKA;1 is constitutively expressed during the cell cycle and likely functions in both S- and M-phase control [47, 48]. It is well-established that CDKA forms a complex with D-type cyclins (CYCD) [36, 49]; and for simplicity, we take this complex to be a single component in the network model. CDKA-CYCD phosphorylates RBR, which inhibits RBR binding to E2F transcriptions factors [50, 51]. This well-known CDKA–RBR–E2F pathway is crucial to the control of the G1/S transition [52].
- **KRP** is a family of CDK inhibitors [53] which bind to CDKA and CYCD to prevent RBR phosphorylation [37, 54].
- **RBR** is a cell-cycle inhibitor. RBR forms a repressor complex with E2F transcription factors to inhibit S-phase entry [55, 56]. We assume that the total amount of RBR is constant over the cell cycle, given observations that RBR transcription is not regulated during cell-cycle progression [57].
- **E2FA** is a member of the E2F family of transcription factors. E2FA regulates the expression of genes at the G1/S transition to mediate DNA synthesis [58]. E2FA promotes the expression of E2FB [59] and FBL17 [37].
- **FBL17** is a component of the SKP-CULLIN-F-BOX complex, which ubiquitinates several members of the KRP family, targeting them for degradation by proteasomes [37, 60, 61].
- **E2FB** is another E2F transcription factor which promotes the G1/S transition and DNA synthesis. Furthermore, targets of E2FB are genes needed for G2/M transition; particularly CYCB1;1, which is induced when RBR-free E2FB increases [62, 63]. Additionally, as E2F1 induces expression of the endogenous Skp2 gene in humans [64] Ortiz-Gutiérrez *et al.* hypothesise that in Arabidopsis E2FB activates Skp, a subunit of SCF complexes [38].

#### G2-M components

- **CDKA/B: CYCB**. CDKB is a plant-specific CDK [34, 65] which is required for normal cell-cycle progression [66], as it promotes the G2/M transition [67]. CDKB can associate with both A- and B-type cyclins (CYCA, CYCB) [44, 68, 69]. Both CDKA and CDKB form a complex with CYCB, and we take active complexes of CDKA/B with CYCB to be a single component in the network model. These complexes phosphorylate MYB transcription factors: MYB3R3 is inhibited and MYB3R4 is activated by phosphorylation [70].
- **MYB3R3** is a transcription factor which regulates genes at the G2/M transition [71, 72]. MYB3R3 is a transcrptional repressor of a set of genes which promote mitosis [73], including CDKB2 and CYCB [70].
- **MYB3R4** is another member of the MYB family. It has a peak of expression at G2/M [74] and acts as a transcriptional activator of genes expressed with critical roles in cytokinesis [74]. CDKB2 and CYCB are activated by MYB3R4 [70]. MYB3R4 promotes the expression of certain subunits of the anaphase promoting complex (APC) [38]. Interestingly, MYB3R4 may exhibit autoactivation. The tobacco genes, NtMYBA1 and NtMYBA2 (which are analogous to Arabidopsis MYB [75]), contain the MSA sequence [76] and thus these genes are possibly activated by their own products [77].
- **SMR** (SIAMESE-RELATED) proteins form a family of plant-specific [71, 78] CDK inhibitors, which inhibit the G2/M transition [79, 80]. SMR1 forms an inhibitory complex with CDK [81].

#### Linking components

- **SCF** complexes target specific proteins for degradation by proteasomes [82, 83]. CYCD, a highly unstable protein, is degraded by a SCF-mediated proteasome-dependent mechanism [84].
- **APC** (anaphase promoting complex) is a multi-subunit complex that targets proteins for degradation via proteasomes [85], particularly Cyclin B [86] and Cyclin A [87].

The above interactions (summarised in Fig 1) are represented by a system of coupled ordinary-differential equations for the concentrations of the twelve components listed above (see Methods section 5 for the model, and its derivation).

### 2.2 Multi-cell population model

The network model outlined in section 2.1 (given as a system of ordinary differential equations in Methods section 5) simulates the progression of a single cell through the cell cycle. Once a specified critical condition is reached, the cell cycle concludes and the cell divides. To understand mechanistically how this cell-cycle network controls cell size within a biological tissue, we will embed this network model into a population of growing and dividing cells, where cell division is governed by the status of the cell-cycle network. Throughout this study, the conditions are as follows:

- E2FA and E2FB transition from a quasi-steady state of low activity to a quasi-steady state of high activity in order to initiate the G1/S transition. E2FA/B is assumed to be inactive when bound by RBR.
- Similarly, MYB3R4 activity transitions from low to high and MYB3R3 activity transitions from high to low in order to initiate the G2/M transition. MYB3R4 which has been phosphorylated (by CDK) is active, and phosphorylated MYB3R3 is inactive.

Unless otherwise stated, we assume that proteins are synthesised at a rate that scales with cell size, as is thought to be appropriate for the majority of proteins [19–22]. In specific simulations, we consider size-independent proteins in which synthesis rates are constant and do not scale with cell size. We note that although DNA replication during S phase doubles the gene copy number, it is thought that this replication does not affect transcription rates (see [88] and references therein), and therefore for all proteins, we assume that the synthesis rates do not depend on the cell-cycle phase.

Once a cell has progressed through both G1/S and G2/M, it divides forming two daughter cells. The volume of the mother cell, and the mass of the proteins contained within, are then distributed to the daughters. We assume that cell division is stochastic such that the proportion of the mother cell’s volume given to one of the daughter cells is given by the random variable, *d*, chosen from a truncated normal distribution with mean $\frac{1}{2}$ on the interval [0, 1]. Thus, if *V*_0_ is the volume of the mother cell at division, then the volumes of the daughter cells at birth are given by *V*_1_ = *dV*_0_ and *V*_2_ = (1 − *d*)*V*_0_.

At division, most proteins are handled similarly. We assume that, after the breakdown of the nuclear envelope at division, diffusion rapidly drives protein molecules towards uniform concentration throughout the cell. Thus, the inherited mass of protein is directly proportional to the volume of the daughter cell such that the protein mass in the two daughter cells are *X*_1_ = *dX*_0_ and *X*_2_ = (1 − *d*)*X*_0_, where *X*_0_ is the protein mass in the mother cell at division.

For some specified proteins, it is assumed that almost all molecules are stably bound to chromatin. Both daughters inherit an equal mass of chromatin at division, and thus inherit an equal mass of protein so that $X_{1}=X_{2}=\frac{X_{0}}{2}$. Such proteins are said to be equally inherited. We explicitly state when we assume a protein is equally inherited, and no proteins are equally inherited unless stated to be.

Once division is complete, the cell-cycle model is simulated in both daughter cells. This process is then iterated, generating a population of theoretical cells. The volume of each cell and the time at birth, G1/S and G2/M are recorded. The relationship between the volume of a cell at birth and its volume at division can be used to predict the efficacy of size control.

#### 2.2.1 Simple example: Sizers, adders and timers

Before progressing with the full cell-cycle model, we first gain intuition into potential cell-size control mechanisms by simulating the the cell population model with highly simplified cell types each corresponding to a phenomenological size-control mechanism. For simplicity, we assume that cells grow exponentially $\dot{V}\left(t\right)=r_{gr}V$. The three simple cell types: sizers, adders and timers [4, 7], function as follows:

- Sizer: an ideal sizer always divides after reaching a fixed size threshold, *V*_*S*_; divide if *V*(*t*) ≥ *V*_*S*_. If a cell divides asymmetrically, the daughter cells nonetheless divide at the same size. In this way, cell-size variation is eliminated in a single cell cycle.
- Adder: an ideal adder grows by a fixed amount, *V*_*A*_ before dividing; divide if *V*(*t*) ≥ *V*(*t*_0_) + *V*_*A*_, where *t*_0_ is the time of birth. Cells which are larger at birth will still end up larger at division than cells which are smaller at birth; however, size variation at division in a population of adder cells decreases over time.
- Timer: timer cells divide after a fixed amount of time, *t*_*c*_ has elapsed since their birth; divide if *t* ≥ *t*_0_ + *t*_*c*_. A timer mechanism establishes size control only if cells exactly double in size between birth and division and cell size is perfectly symmetrical. Any perturbation away from perfect volume doubling and division symmetry will cause the variation in cell size to grow over time.

**Fig 2:**
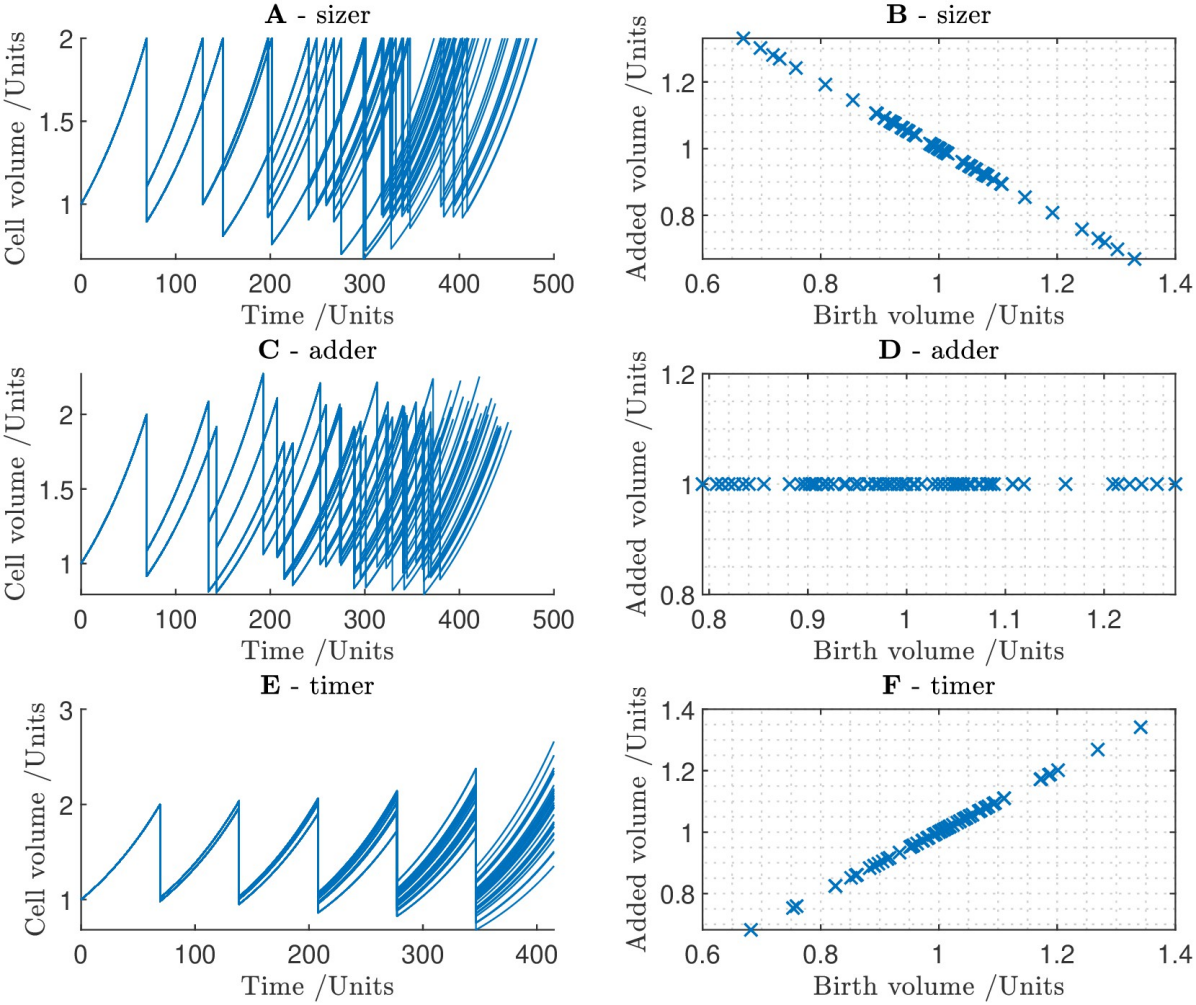
Simulations cell division within a cell population reveal how simple cell-division rules affect the cell-size distribution. The simulation begins with a single cell of unit volume, which divides to produce daughter cells. Cells grow exponentially until the specified division condition is satisfied. Cell division is stochastic; at each cell division, a normally distributed random variable is generated to determine the proportion of the cell volume distributed to the daughter cells. **A** Time course for ideal sizers. **B** Scatter plot of cell volume at birth against added volume at division (volume at division minus volume at birth) for ideal sizers. **C** Time course for ideal adders. **D** Scatter plot for ideal adders. **E** Time course for ideal timers. **F** Scatter plot for ideal timers.

Time courses of populations of simulated cells of these three idealised types are presented in Fig 2 (A, C, E for sizer, adder and timer respectively), as are scatter plots of the relationship between cell volume at birth and added volume during one cell cycle (B, D, F for sizer, adder and timer respectively). Sizer and adder cells divide asynchronously but maintain cell-size control over successive generations. Timer cells divide simultaneously, but larger cells add more volume before division than smaller cells, causing the range of cell size to diverge over time. The failure of a timer mechanism to establish cell-size control shows that the cell cycle cannot function merely as a ‘molecular clock’. Cell-cycle progression must be coupled to cell size in some way, as a lack of size control would ultimately prove fatal to the organism.

For any given population of cells (real or theoretical), we can plot the relationship between cell volume at birth and added volume at division. The gradient of this line (or curve, in general) is enough to classify the cells. If the gradient is −1, the cells are perfect sizers. If the gradient is between −1 and 0 the cells are imperfect sizers. If the gradient is 0, the cells are adders. As long as the gradient is non-positive, cell size homeostasis is assured. If the gradient is positive, cell size control will not be maintained.

Thus, these simulations of simple cell types have revealed that either adder or sizer type behaviour for cell division can lead to cell-size control being achieved within a cell population. Our results below reveal how such behaviour can be created mechanistically via the cell-cycle network.

## 3 Simulation results

### 3.1 Full network model exhibits limit cycles

**Fig 3:**
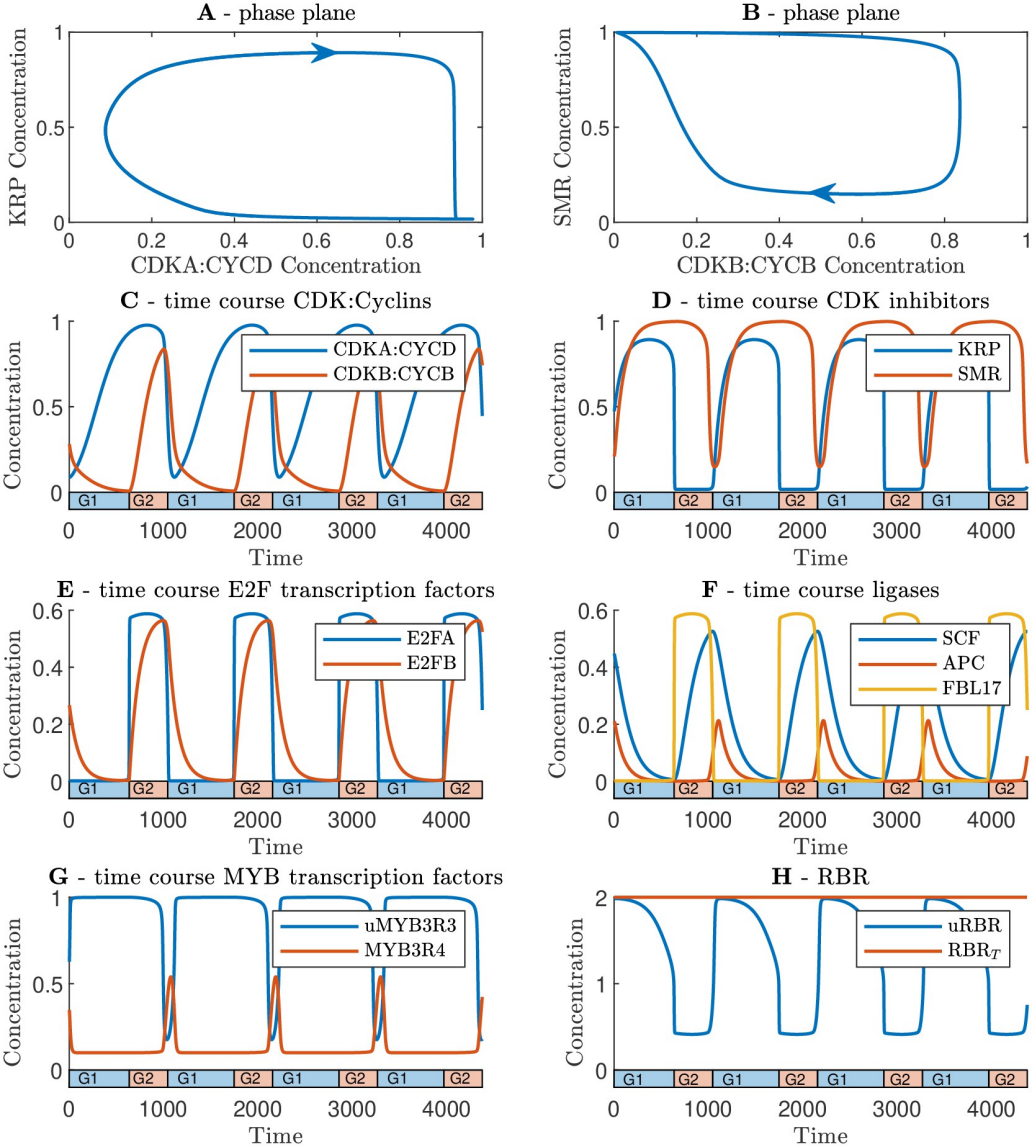
Simulations of the full cell-cycle model reveal limit-cycle dynamics. **A** phase plane orbit showing CDKA and KRP. **B** phase plane orbit showing SMR and CDKB. **C**-**H** Time course for each protein as indicated. We note that in panel H, we show both total RBR (RBR_*T*_) and unphosphorylated RBR (RBR_*u*_). Parameter values used in the simulations are given in Methods section 5.5 with the exception of the cell relative growth rate which is set to *r*_*gr*_ = 0 for this simulation.

We first simulate the network equations in terms of the concentrations in a single cell (Fig 3). These simulations demonstrate that the network exhibits limit-cycle solutions (Fig 3A and 3B), with accumulation of G1/S- and M-phase cyclins driving the cell cycle forward (Fig 3C). Simulations were performed using the parameter values described in Methods section 5.5. We verified that limit cycles still occur for ranges of each parameter choice (albeit noting that a few parameter values are particularly constrained, such as the CDKA:CYCD synthesis rate which cannot be too small as CDKA:CYCD is required to activate the G1/S transition) (see Methods section 5.5).

The predicted dynamics are consistent with published data available for several components. The model predicts the well-documented bursts of CYCB-associated activity in late G2 phase [89], the G2 phase-specific expression of FBL17 observed in [14], and the peak of MYB3R4 at G2/M observed in [74].

The degradation of the cyclins by ubiquitin ligases (SCF and APC for CYCD and CYCB respectively) establishes a limit cycle by resetting the cyclins to the original state [90]. For the limit cycle to occur, it is vital that this degradation occurs via a delay mechanism. If, for example, SCF were activated directly by E2FA, the system would reach a stable equilibrium. However, because there is a delay period in which E2FB accumulates before SCF expression is unleashed, then a cyclical solution is produced. Similarly at G2/M, this delay is established by the need for MYB3R4 to accumulate before APC complexes can be produced.

### 3.2 Network exhibits biological switch governing G1/S

In this section, we isolate the network dynamics at the G1/S transitions in order to demonstrate that biological switches are a genuine property of the model at this transition. To do this, we solve just the G1/S Eqs (25)–(36) in isolation (Methods section 5). We set the mass of other proteins to 0, *i.e.* CDKA/B : CYCB_*T*_ = MYB3R3_*T*_ = MYB3R4_*T*_ = SMR_*T*_ = FBP = SCF = APC = 0.

**Fig 4:**
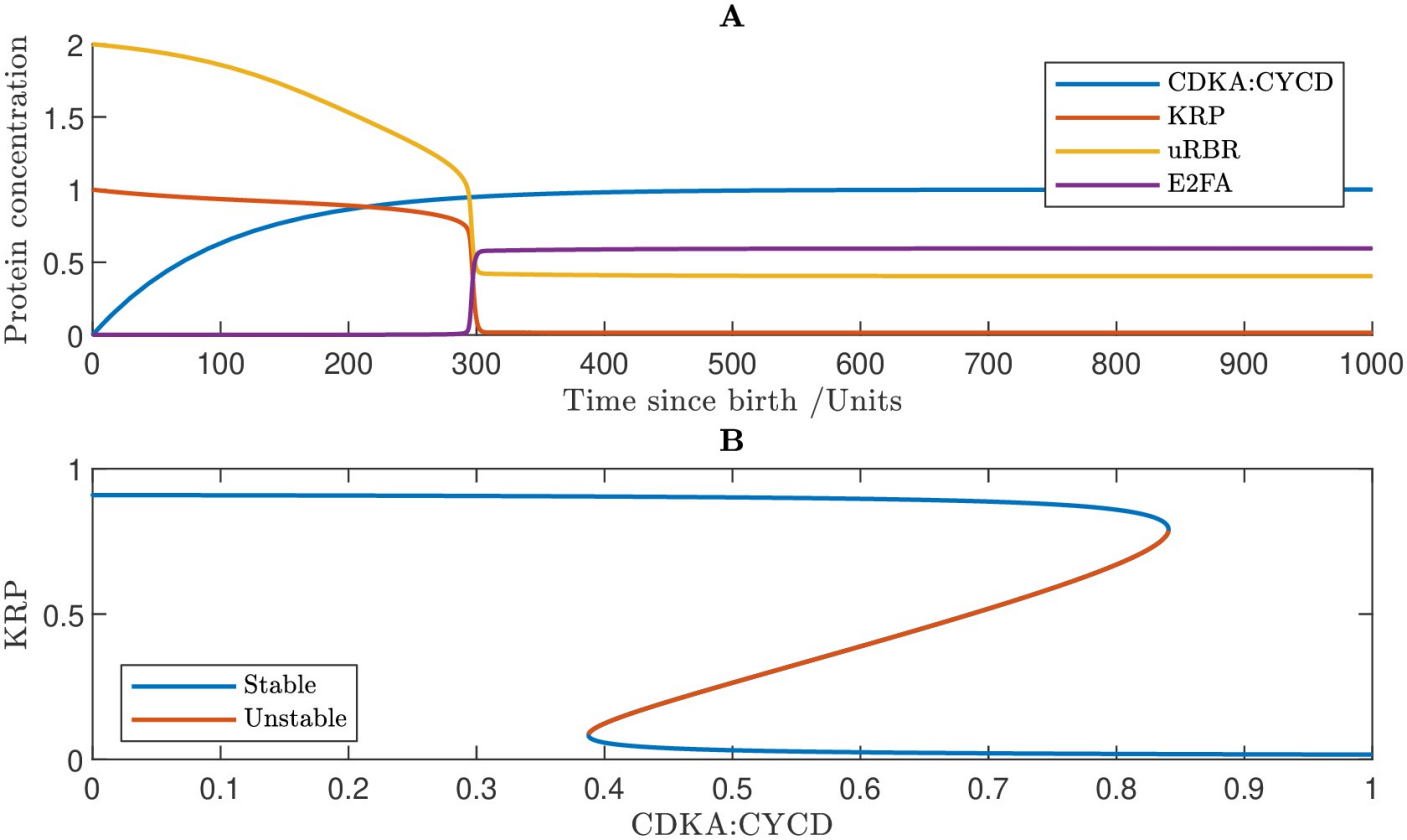
Modelling reveals that the network exhibits a hysteresis at the G1/S transition. **A** Simulated time course showing the concentrations of CDKA, KRP, un-phosphorylated RBR (uRBR) and active E2FA. The transition is clearly visible at the point when E2FA is suddenly released from RBR inactivation. **B** Bifurcation diagram showing KRP steady states. At low levels of CDKA, there exists a unique stable steady state of high KRP activity. Eventually, this state is eliminated in a saddle node bifurcation, and the system switches to a new stable state of low KRP activity. Specifically, (36) subject to CDKA/B : CYCB_*T*_ = MYB3R3_*T*_ = MYB3R4_*T*_ = SMR_*T*_ = FBP = SCF = APC = 0. Parameter values are given in Methods section 5.5.

In Fig 4 it can be observed that the model exhibits hysteresis at the G1/S transition. This is caused by the closed loop of mutual inhibition between cell-cycle activators (CDKA:CYCD, E2FA) and repressors (KRP and RBR), creating a bistable switch. Initially, KRP and RBR are dominant, and E2FA is inactive. As CDKA:CYCD accumulates, this stable state is eliminated and the system switches to a new stable state in which E2FA activity is high. Under the assumption that KRP is rapidly degraded due to FBL17, the state change is sudden. We note that similar G1/S switch behaviour has previously been predicted in the network model of Zhao *et al.* [37], albeit modelling five distinct members of the KRP family.

### 3.3 Network exhibits biological switch governing G2/M

The G2/M transition is much less well understood in plants than G1/S. We hypothesise that the collection of proteins known to be involved in the regulation of G2/M produces a bistable switch, comparable to the switch at the G1/S; we use our model to demonstrate that the network exhibits this behaviour. We consider the G2/M Eqs (37)–(49) in isolation, setting CDKA : CYCD_*T*_ = KRP_*T*_ = E2FA_*T*_ = E2FB = RBR_*T*_ = FBL17 = SCFAPC = 0.

**Fig 5:**
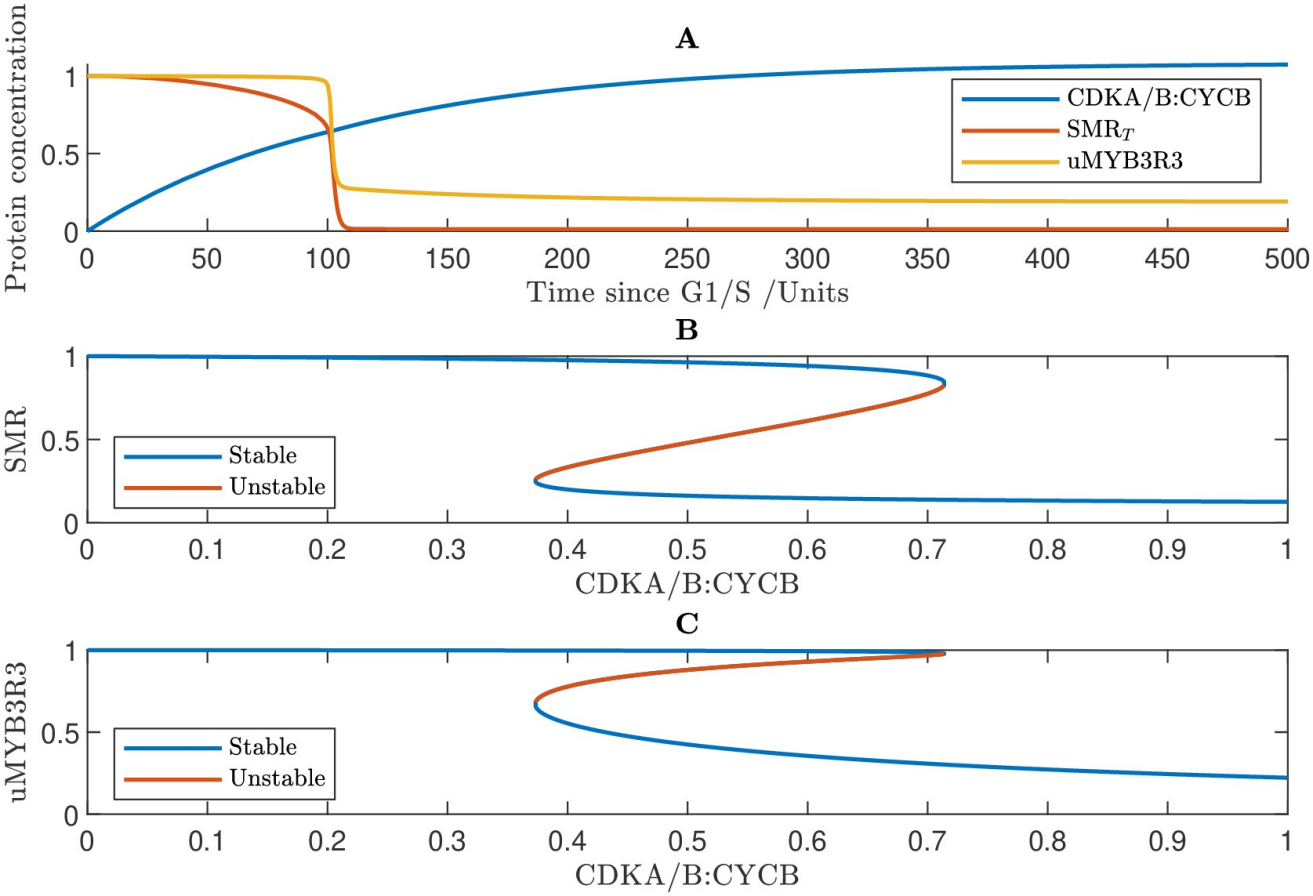
Modelling reveals that the network exhibits a hysteresis at the G2/M transition. **A** Simulated time course showing the concentrations of CDKB, SMR and un-phosphorylated MYB3R3 (uMYB3R3). **B** Bifurcation diagram showing SMR steady states. **C** Relationship between uMYB3R3 concentration and CDKA/B:CYCB concentration. Results are based on numerical simulations of the G2/M Eqs (37)–(49) subject to CDKA : CYCD_*T*_ = KRP_*T*_ = E2FA_*T*_ = E2FB = RBR_*T*_ = FBL17 = SCFAPC = 0. Parameter values used in the simulations are given in Methods section 5.5 with the exception the synthesis rate of CDKA/B:CYCB is *r*_*cb*_ = 0.01 (default value being 0) so that CDKA/B:CYCB is expressed independently of the E2FB transcription factor.

Similarly to G1/S, mutual inhibition between activators and repressors establishes bistability at the G2/M transition (Fig 5). Unphosphrylated SMR binds with CDKA/B:CYCB and inhibits its activity; however, CDKA/B:CYCB accumulation increases SMR phosphorylation. The model predicts that this CDKA/B:CYCB—SMR feedback results in a biological switch. The bifurcation diagram shows that at low levels of CDKA/B:CYCB, there exists a unique stable steady state of high SMR activity; however, as CDKA/B:CYCB increases, this steady state is eliminated in a saddle node bifurcation, and the system switches to a new stable state of low SMR activity (Fig 5B). CDKA/B:CYCB accumulation also increases MYB3R3 phosphorylation, leading to a reduction in the active unphosphorylated form of MYB3R3. The model predicts that the bistability created by the mutual inhibition between SMR and CDKA/B:CYCB leads to a hysteretic relationship between CDKA/B:CYCB and unphosphorylated MYB3R3 (Fig 5C). We note that simulating the interactions between CDKA/B:CYCB and MYB3R3 in isolation, reveals that despite their mutual inhibition, their relationship is monostable, suggesting that SMR is essential in establishing the G2/M switch (see Methods section 5.4).

### 3.4 Model predicts that either size-independence or equal inheritance of inhibitor proteins can lead to size control at G1/S

Having studied the cell-cycle network in isolation, we now investigate the dynamics in a growing cell. This enables us to study how properties of the cell-cycle network (and its components) could enable the G1/S and G2/M transitions to depend on cell size. We aim to identify mechanisms whereby larger daughter cells progress through the cell cycle more rapidly, which would reduce variations in cell size. We focus on two potential size-control mechanisms: (i) interactions between size-independent inhibitor proteins and size-dependent activator proteins and (ii) equal inheritance of inhibitor proteins. Previous sections supposed that all the proteins are synthesised in a size-dependent manner (with the synthesis rates scaling with cell volume). However, if certain inhibitor proteins are size independent then this may give rise to cell-size control (as with a constant synthesis rate, the protein concentration will depend on cell size due to growth-induced dilution). Similar size-control mechanisms have been proposed to control the G1/S transition in budding yeast [15]. In addition, in plants an alternative mechanism has recently been proposed, with size control of the G1/S transition being achieved via the inhibitor protein KRP being equally inherited by two daughter cells [14]. The network model developed in the previous sections enables us to investigate how these mechanisms influence cell-cycle progression.

To investigate how cell size control can be established as an emergent property of the protein regulatory network, we simulate the network model within growing cells, considering cells of varying initial volumes and protein masses (see Methods section 5.3). Unless otherwise stated, we assume that all proteins are size-dependent, and that the initial mass of each protein is proportional to the initial volume of the cell. For G1/S, we test four hypotheses:

- 0. All proteins are size-dependent
- 1. Equal inheritance of KRP
- 2. RBR is size-independent
- 3. KRP is size-independent

Hypothesis 1 is essentially the theory of D’Ario *et al.* that cells inherit an equal mass of KRP regardless of size [14]. The justification for hypothesis 2 RBR is an ortholog of the yeast protein Whi5 [26, 91], which has been observed to be size-independent [20], hence RBR is a good candidate for a size-independent protein. With regard to hypothesis 3, as KRP is a CDK inhibitor, it is a theoretically possible candidate for a size-independent protein.

**Fig 6:**
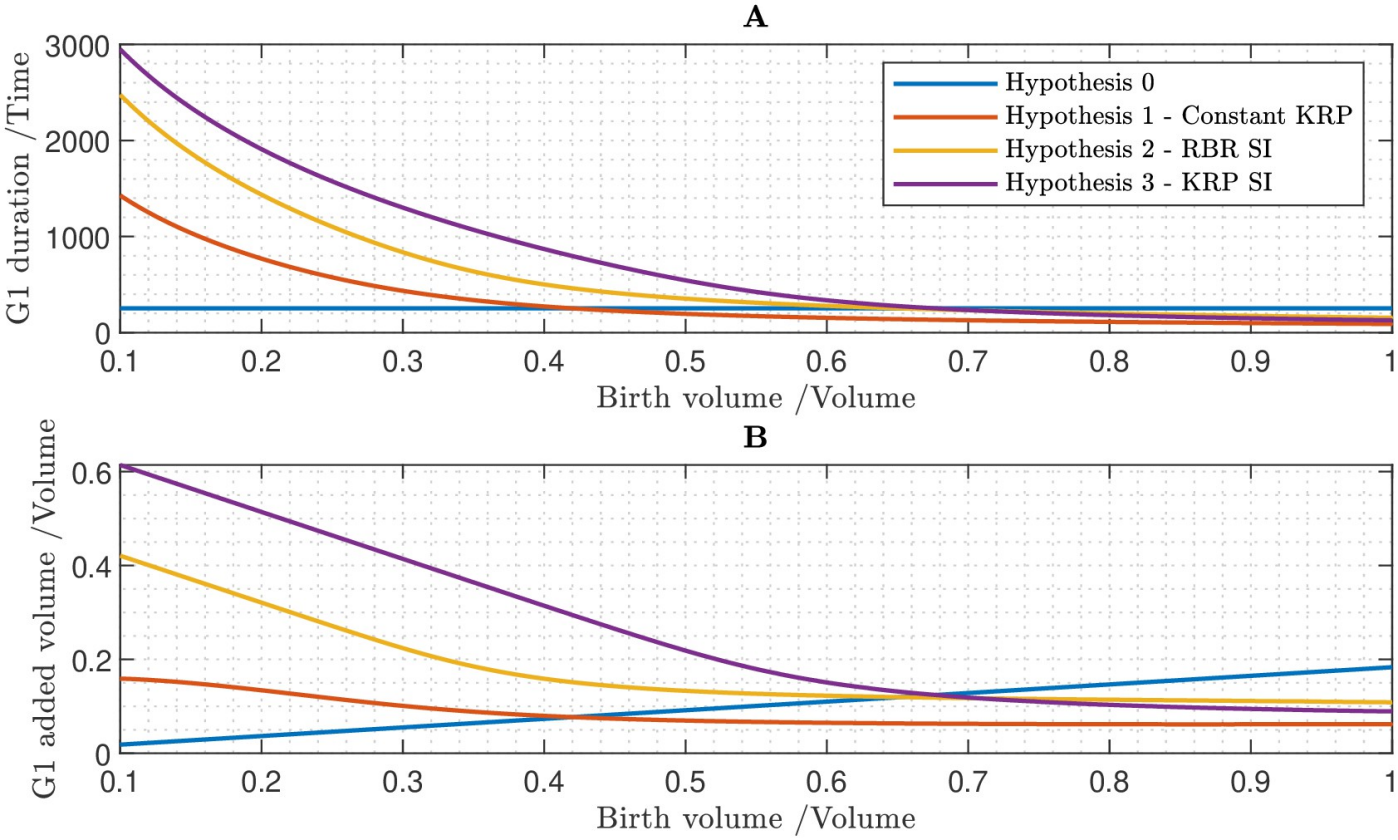
Cell size control at the G1/S transition, testing 4 hypotheses: 0. All proteins are size-dependent. 1. All proteins are size-dependent, but cells inherit a fixed mass of KRP at birth, irrespective of cell volume at birth. 2. RBR is size-independent and all other proteins are size-dependent. 3. KRP is size-independent and all other proteins are size-dependent. **A** Time between birth and S phase (*i.e.* the duration of G1 phase) plotted against birth volume. **B** Cell volume growth during G1 phase.

Results in Fig 6 indicate that hypothesis 0 (all proteins are size-dependent) fails to control cell size, as there is a positive relationship between cell volume at birth and added volume during G1 phase. If either KRP or RBR are size independent then there is an overall negative relationship between birth volume and added volume (thus, this mechanism would mitigate variations in birth size). Interestingly, the trend is steeper for cells of smaller birth volume suggesting that smaller cells behave more like imperfect sizers, while larger cells behave more like adders. It is also interesting that either KRP or RBR being size independent would contribute to cell-size control, despite their differing functions. The equal inheritance of KRP also successfully establishes size control, although in this case the trend curve relating birth volume and added volume is far shallower, suggesting that the behaviour of the cell in this case is overall closest to an adder.

### 3.5 Equal inheritance or size-independence of SMR predicts size control at G2/M

There is empirical evidence that plants exhibit separate size control mechanisms at G1/S and G2/M [13]. We now consider the latter case. Within our model, there are two proteins which inhibit CDKA/B:CYCB (and hence G2/M): SMR and MYB3R3. We investigate whether size independence and equal inheritance of such inhibitor proteins can account for size control at G2/M. We examine four hypotheses:

- 0. All proteins are size-dependent
- 1. Equal inheritance of SMR
- 2. SMR is size-independent
- 3. MYB3R3 is size-independent

**Fig 7:**
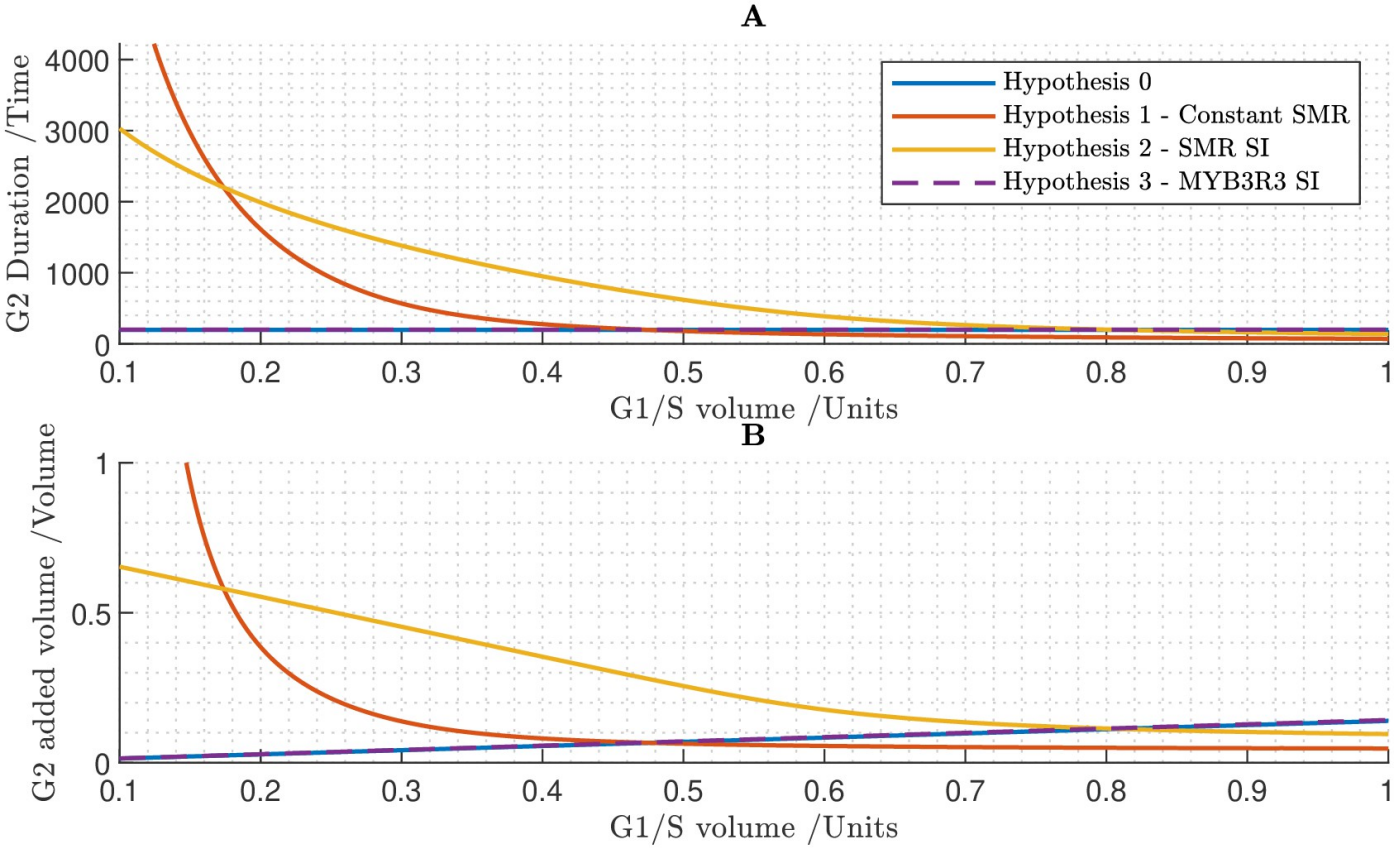
Cell size control at the G2/M transition, testing 4 hypotheses: 0. All proteins are size-dependent. 1. All proteins are size-dependent, but cells inherit a fixed mass of SMR at birth, irrespective of cell volume at birth. 2. SMR is size-independent and all other proteins are size-dependent. 3. MYB3R3 is size-independent and all other proteins are size-dependent. **A** Time between S phase and M phase (*i.e.* the duration of G2 phase) plotted against birth volume. **B** Cell volume growth during G2 phase.

Results for G2/M are displayed in Fig 7. Unsurprisingly we find no basis for size control if all proteins are size dependent. We find that both size independence and equal inheritance of SMR establish size control. Size independence of MYB3R3 produces results no different from the scenario in which all proteins are size dependent. Although MYB3R3 is a cyclin inhibitor, the model predicts that this by itself is insufficient for the size independence of MYB3R3 to yield size control. We note that while MYB3R3 is in a double negative feedback loop with CDKA/B:CYCB, these interactions lead to a monostable relationship between these components (see Methods section 5.4). Thus, we surmise that MYB3R3 modulates the expression of CDKA/B:CYCB rather than contributing directly to the bistability and size control.

### 3.6 Full cell cycle demonstrates that size-independent inhibitor proteins enables cell population to maintain size homeostasis

To fully test the mechanisms of cell-size control, we now investigate these within a cell population. We present results following the methods outlined in section 2.2 to simulate the entire cell cycle iterated over generations. Unlike the results in sections 3.4 and 3.5, which isolate G1/S and G2/M respectively in a single cell cycle, the simulations in this section encompass the full cell-cycle model iterated over multiple generations.

**Fig 8:**
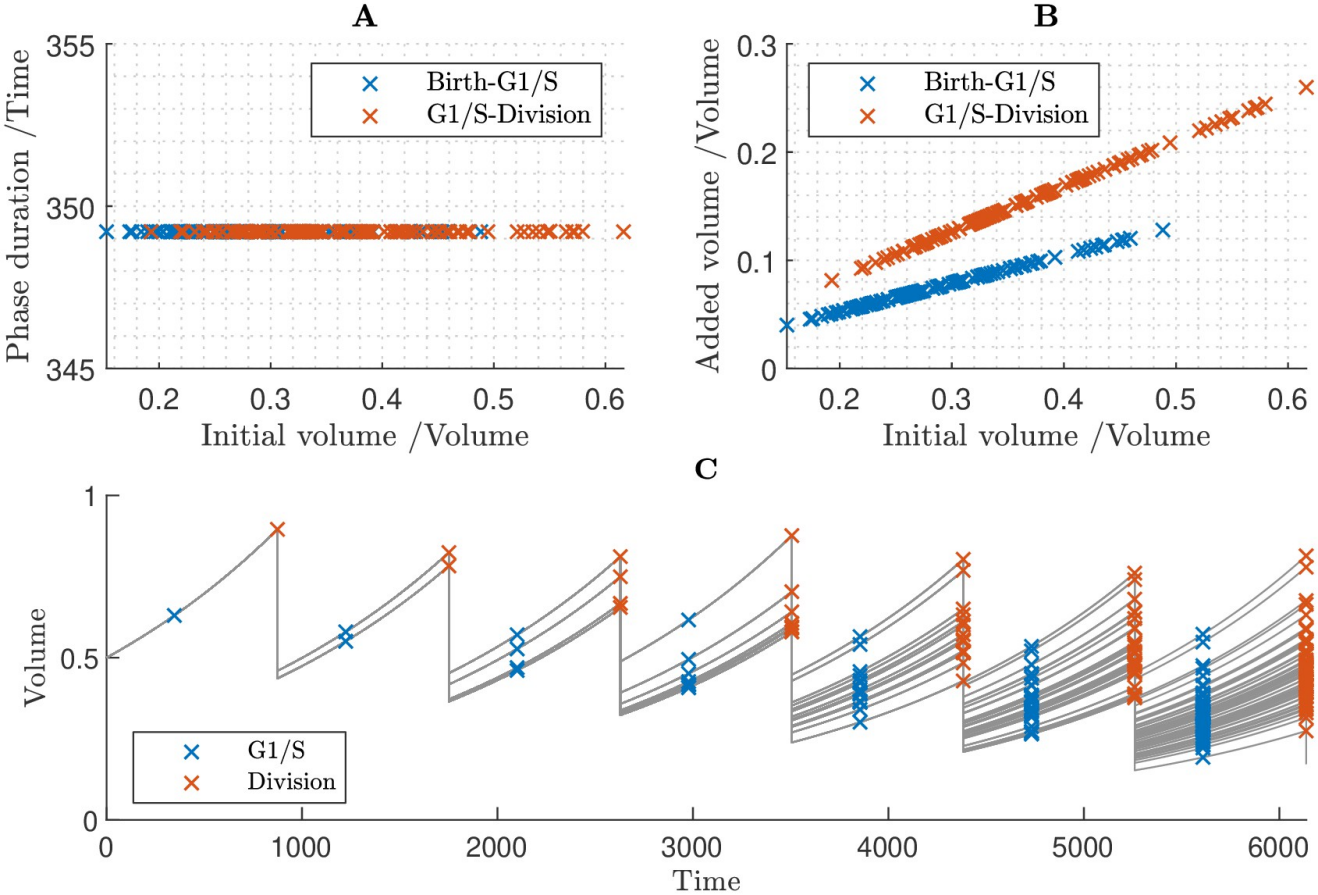
Cell population simulation under the assumption that all proteins are size-dependent, which fails to produce size control. **A** Relationships between G1 duration and birth volume, and between G2/M duration and volume at the G1/S transition. **B** Relationships between added volume during G1 and birth volume, and between added volume during G2/M and volume at the G1/S transition. **C** Time course of cell population volumes.

In the simulations shown in Fig 8 we assumed that all proteins are size-dependent. Under this assumption, all proteins accumulate in fixed proportion to one another. This assumption yields an almost perfect timer mechanism in which cells divide after a fixed interval of time, and hence cells divide synchronously across generations. Consistent with the results of sections 3.4 and 3.5, this fails to control cell size.

**Fig 9:**
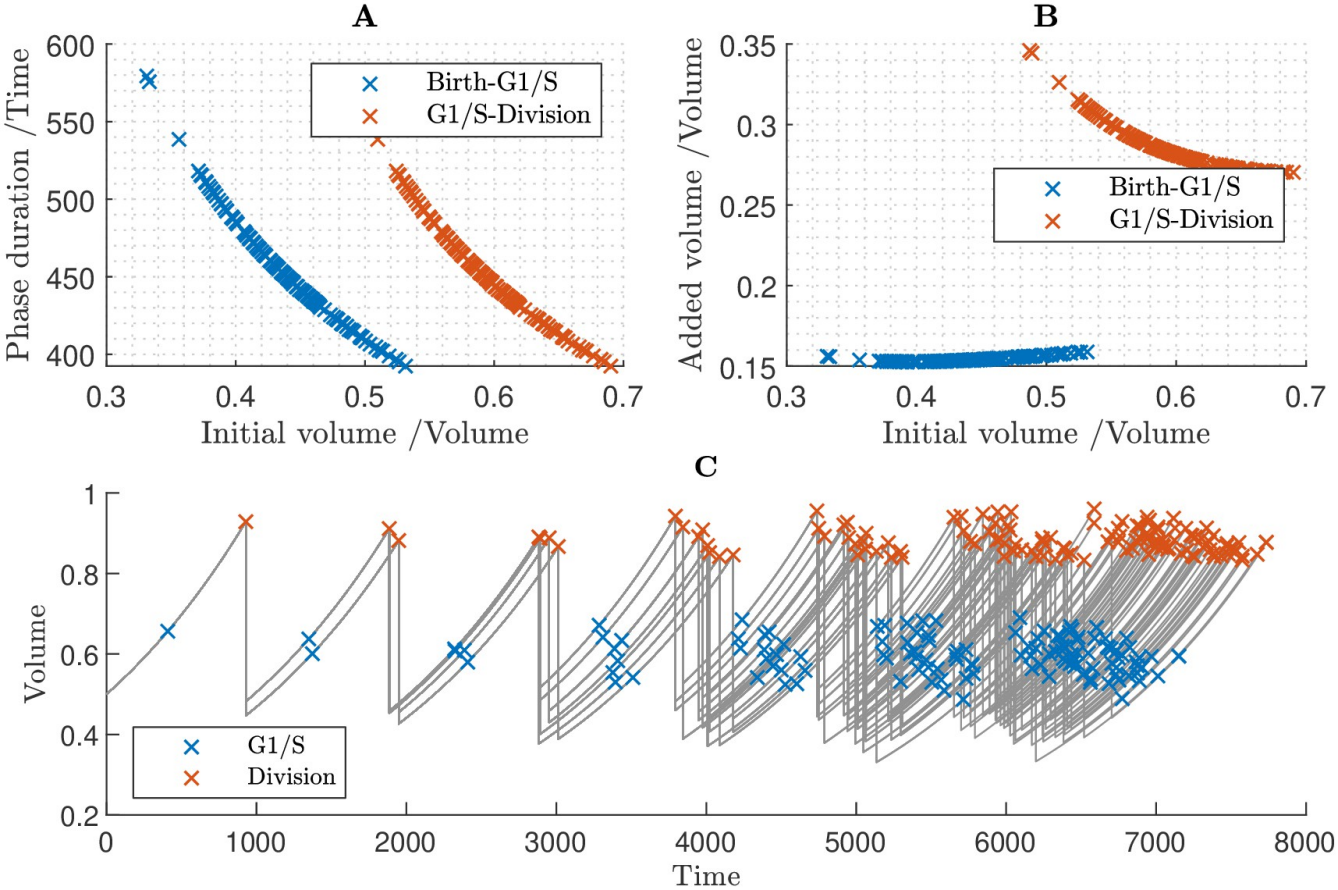
Cell population simulation under the assumption that all proteins are size-dependent except KRP and SMR, which are size-independent. Successful cell size homeostasis is established. **A** Relationships between G1 duration and birth volume, and between G2/M duration and volume at the G1/S transition. **B** Relationships between added volume during G1 and birth volume, and between added volume during G2/M and volume at the G1/S transition. **C** Time course of cell population volumes.

In Fig 9, we assumed that all proteins are size-dependent except the S-phase CDK inhibitor KRP and the M-phase CDK inhibitor SMR, which are set to be size-independent. Under this set of assumptions, there are negative relationships both between cell birth volume and the duration of G1 and between the cell volume at the G1/S transition and the duration of S/G2/M (Fig 9A). Hence, both cell-cycle phases contribute to size control: larger cells divide sooner, having added less volume, than smaller cells, and our model predicts size homeostasis over multiple generations (Fig 9C). That size-independence of KRP and SMR provides size control during both G1/S and G2/M is consistent with the findings in sections 3.4 and 3.5; however, we note that here G1 duration is predicted to be independent of birth volume (Fig 9B), suggesting that the cell sizes at birth are sufficiently large that we are in the large-cell ‘adder’ regime shown in Fig 6. In summary, these results predict that smaller cells behave like imperfect sizers, while larger cells behave more like adders, resulting in cell-size homeostasis being maintained across generations.

To quantify how size independence of KRP and SMR affect the distribution of cell sizes, we calculated the quartile co-efficient of dispersion for the cell sizes at key points in the cell cycle (calculating these from the cell-size values for all simulated cells) (see Table 1). We see that this dispersion is reduced to 75% of the birth value by the G1/S transition, and to 36% of the birth value by the G2/M transition, showing that cell size is controlled via a reduction in the dispersion of cell sizes during both G1/S and G2/M. We note that we find similar reductions in the dispersion of the cell sizes over both the G1/S and G2/M phases with alternative choices of the parameter values (see Methods section 5.5 and S1 Table).

**Table 1:** Quartile coefficient of dispersion, (*Q*_3_ − *Q*_1_)/(*Q*_3_ + *Q*_1_), at birth, G1/S and G2/M for different size control hypotheses. Abbreviations are: “Eq Inh” = “Equal inheritance”; “SI” = “size-independent”; “SD” = “size-dependent”; “PD” = “phase-dependent” *i.e.* the synthesis of KRP is restricted to M-phase due to being limited by MYB3R4 activity. “X:Y” means the ratio of the coefficients of dispersion. Values are calculated from all cells that have completed a full cell cycle within the simulated time.

Hypothesis	Birth	G1/S	G2/M	G1/S:Birth	G2/M:G1/S	G2/M:Birth
KRP SI, SMR SI	0.076	0.055	0.027	0.71	0.50	0.36
KRP Eq Inh, SMR SI	0.071	0.066	0.021	0.93	0.32	0.30
KRP Eq Inh (PD), SMR SI	0.095	0.077	0.029	0.80	0.38	0.31
KRP Eq Inh (PD), SMR SD	0.29	0.22	0.22	0.75	1.00	0.75

### 3.7 Equal inheritance of KRP at division can partially control size at the G1/S transition

Having established that size-independence of inhibitor proteins would be one mechanism to control cell size within a population, we now consider the alternative hypothesis of D’Ario *et al.* that equal inheritance of KRP at division enables the G1/S transition to depend on cell size [14]. To investigate how simulations with this equal-inheritance mechanism compare to those with size-independent KRP (from §3.6), we prescribed equal inheritance of KRP together with size-independence of SMR (providing a size-control mechanism at G2/M). In simulating the equal inheritance, we assume that equal quantities of KRP are inherited by each daughter cell on division (irrespective of cell size). D’Ario et al [14] suggest that this occurs due to KRP binding to the chromatin (that splits equally between the daughter cells), and that any unbound KRP is degraded prior to division. Here, for simplicity, we suppose that the unbound proportion of KRP is sufficiently small, that we can assume all the KRP molecules are divided equally between the two cells.

**Fig 10:**
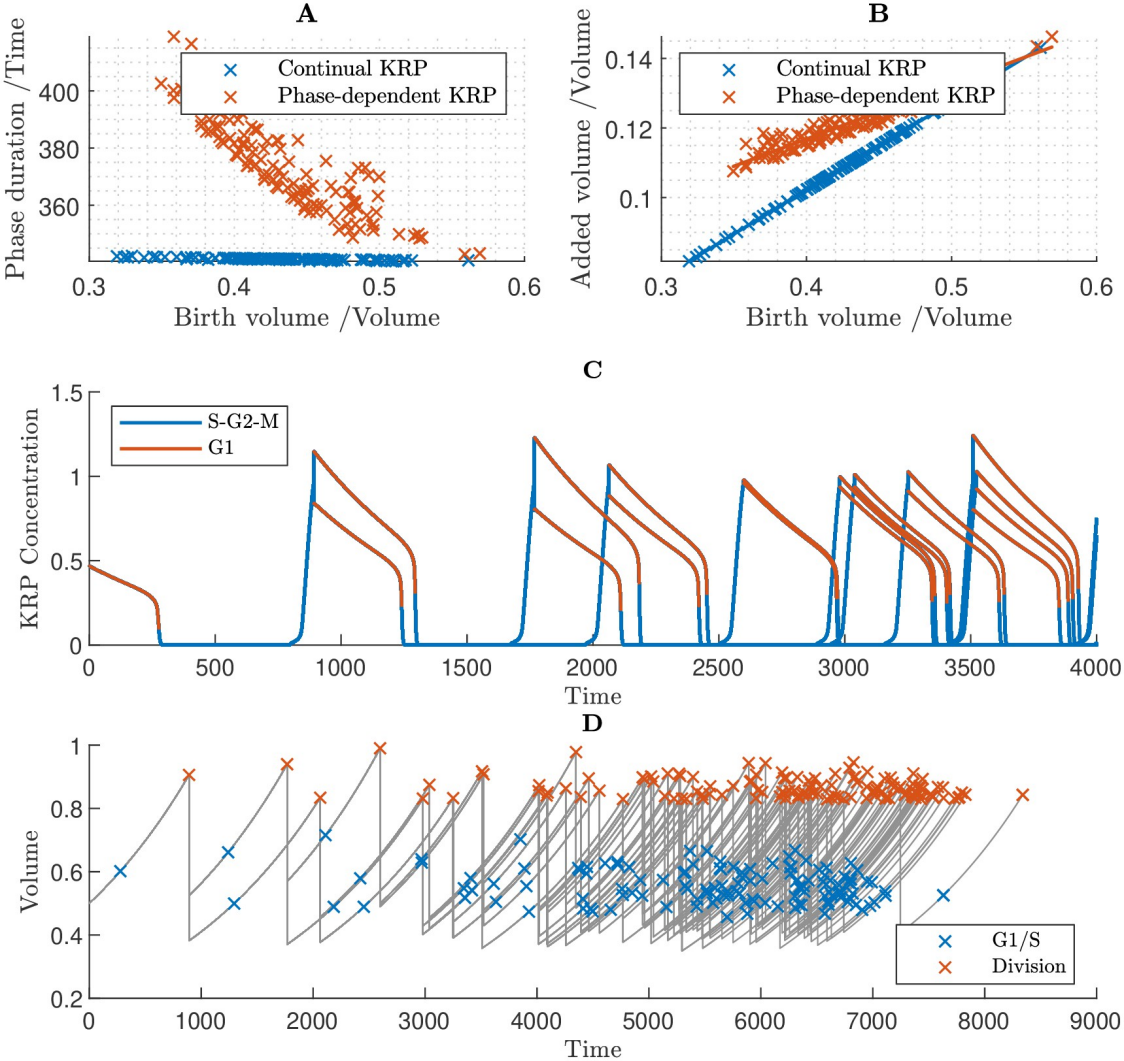
Cell population simulations under the assumptions that KRP is equally inherited, SMR is size independent and all other proteins are size dependent. Simulations considered two scenarios: one in which there is continual KRP synthesis at a constant rate, and one in which KRP synthesis is phase-dependent. In the latter case it is assumed that the synthesis of KRP is proportional to the concentration of MYB3R4, *i.e.* $r_{krp}=0,r_{krp}^{myb4}=0.05$. **A** The duration of G1 phase against birth volume taken from generated cell populations. The Continual KRP synthesis model is in effect a timer mechanism, as the G1 duration is a constant regardless of cell birth volume. In contrast, in the phase-dependent KRP model, larger cells generally have shorter cell cycles in general, which will theoretically establish a degree of size control at G1/S. **B** Added volume during G1 phase. In both models the overall trend is positive, although it is less steep in the case of phase-dependent KRP synthesis. **C** Time course of KRP concentrations, assuming phase-dependent KRP synthesis. KRP is synthesised in late G2/M-phase when MYB3R4 is active, diluted during G1-phase, then rapidly degraded at G1/S. Note that the equal inheritance of KRP mass, causes unequal inheritance of concentration. **D** Time course of cell population volumes, assuming phase-dependent KRP synthesis.

We simulated two versions of this model (Fig 10). In the first instance, we assumed a continual, constant rate of KRP synthesis throughout the cell cycle (as in the previous simulations). Secondly, we imposed conditions such that KRP synthesis is dependent on the cell-cycle phase: we set the base rate constants for KRP synthesis and degradation to 0, and introduced an additional regulatory link, assuming that KRP synthesis is promoted by the MYB3R4 transcription factor [38]. In this way, KRP synthesis would be limited to late G2/M and early G1 phases, consistent with the experimental findings of D’Ario *et al.* [14].

We hypothesised that constant synthesis and degradation would drive the KRP concentration in the daughter cells towards equilibrium during the G1 phase, which would undermine the equal inheritance mechanism and prevent size control. Simulation results are consistent with this hypothesis. Simulations with continual KRP synthesis suggested that G1/S duration does not depend on the cell’s birth size (Fig 10A) and revealed a positive relationship between cell volume at birth and added volume up to G1/S (Fig 10B), which implies that cell-size homeostasis is not maintained.

Simulating the second case in which KRP synthesis is assumed to depend on MYB3R4, we found that the specified assumptions did indeed result in KRP concentrations depending on both the cell-cycle phase and birth size (Fig 10C): rapid synthesis of KRP at the end of G2/M (due to MYB3R4 promoting KRP synthesis) leads to a significant increase in KRP; at cell division, the KRP molecules are divided equally between the two cells, leading to the larger daughter cell having a lower concentration than the smaller daughter cell; during G1, there is no KRP synthesis and dilution leads to a gradual reduction in KRP concentration, until the biological switch at G1/S occurs and the sudden increase in FBL17 rapidly degrades the remaining KRP concentration. Despite the birth-size-dependent KRP concentrations at G1/S, surprisingly, we still observe a positive relationship between birth volume and added volume during G1 (Fig 10B). Nonetheless, the gradient of the line of best fit is more shallow in this case (0.17 with phase-dependent KRP synthesis versus 0.25 with continual KRP synthesis; Fig 10B) which suggests that size homeostasis has been partially, though not entirely, established at G1/S.

Calculating the quartile coefficient of dispersion of the cell sizes at the different points of the cell cycle (Table 1), we found that equal inheritance of KRP reduced the dispersion in cell sizes at the G1/S transition to 80% of that at cell birth, and with size-independent SMR providing further size control of G2/M, the overall reduction in the dispersion of the cell sizes between birth and division is 31%, which is similar to that for the case of KRP being size independent (described in §3.6). Interestingly, even in the case of continual KRP synthesis, which leads to a relatively small reduction in cell-size dispersion at G1/S, the size-control at G2/M is sufficient to compensate and lead to an overall 30% reduction in the cell-size dispersion across the cell cycle.

**Fig 11:**
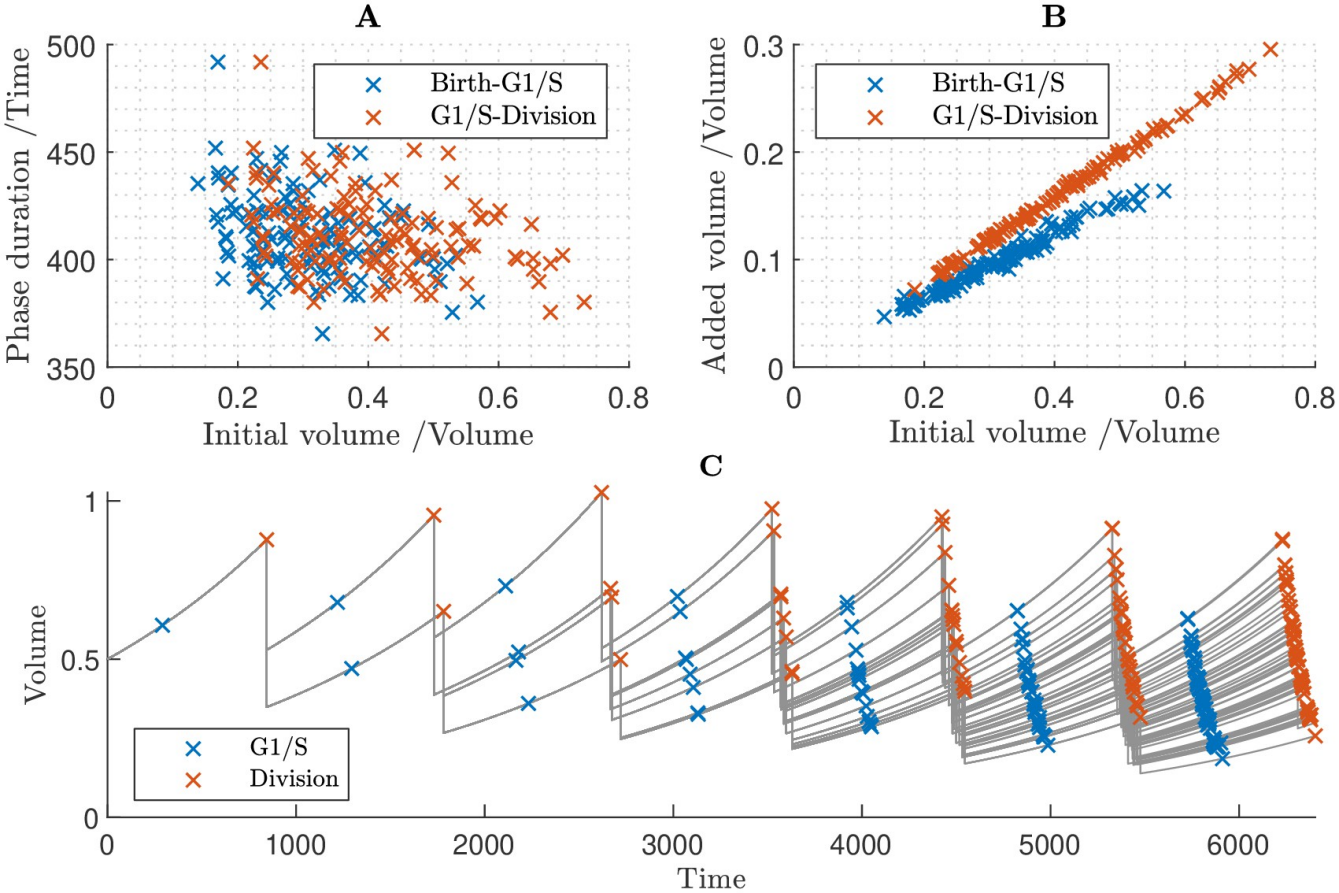
Cell population simulation under the assumption that all proteins are size-dependent, but that KRP is equally inherited and has phase-dependent synthesis (due to regulation by MYB3R4). **A** Relationships between G1 duration and birth volume, and between G2/M duration and volume at the G1/S transition. **B** Relationships between added volume during G1 and birth volume, and between added volume during G2/M and volume at the G1/S transition. **C** Time course of cell population volumes.

To further test the role of size control at the two transitions and the equal inheritance mechanism, we simulated the model with equal inheritance of KRP, and no other size-control mechanism (i.e. assuming all proteins are size dependent) (Fig 11). We found that while equal inheritance of KRP provides partial size-control of G1/S transition, with dispersion of cell sizes at G1/S reducing to 75% of that at birth (Table 1), there is still a positive relationship between birth volume and added volume at G1/S (Fig 11B). The model predicts that after multiple generations the cell population exhibits an increasingly wide range of cell sizes and size homeostasis is not achieved (Fig 11C).

These modelling results suggest that although equal inheritance of KRP contributes to size control, it would need to act in conjunction with some other mechanism controlling G2/M (such as size-independence of SMR) to establish cell-size homeostasis within a cell population.

### 3.8 Mutant phenotypes

As a further test of the model, we simulated mutant phenotypes which have been examined in the empirical literature. We simulated mutations of two proteins, Cyclin D and CDKB, as both of these mutants have been tested by Jones *et al.* [13]. We considered three cases for each protein: wild-type expression, over-expression and under-expression. For wild-type expression, we used default parameter values (given in Methods section 5.5). In the case of over-expression we separately increased the synthesis rates of CDKA:CYCD and CDKA/B:CYCB (*r*_*ca*_ and *r*_*c*_*b*) by 50%. In the case of under-expression we separately decreased *r*_*ca*_ and *r*_*cb*_ by 20%. We simulated the cell population model under the assumption that KRP and SMR are size-independent, as this gives the most robust cell size control and we therefore expect mutations to have the strongest effect on cell size in this case.

**Fig 12:**
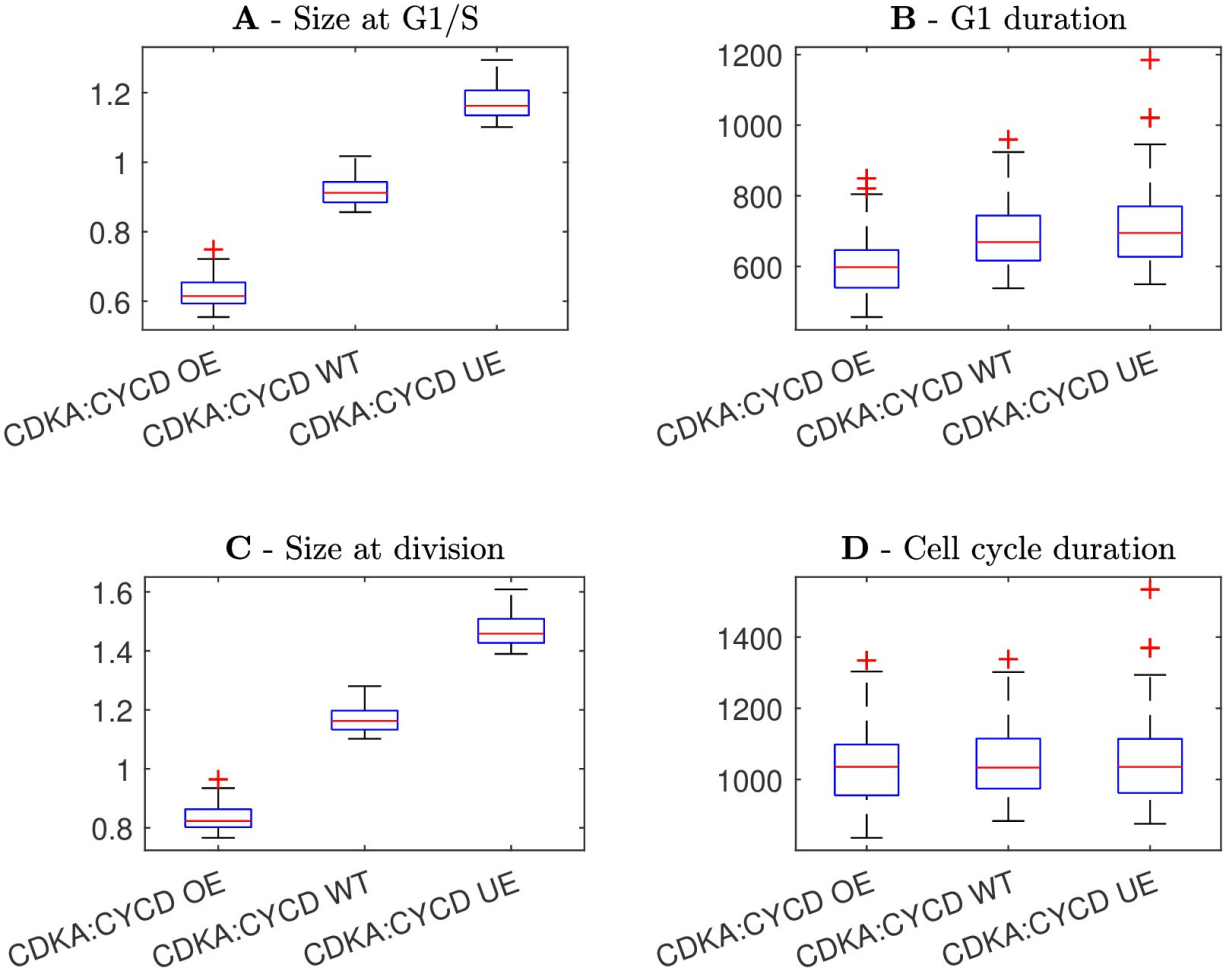
Box plots showing the distribution of cell sizes and phase durations under the assumption of CDKA:CYCD over-expression (OE), wild-type expression (WT), and under-expression (UE). Wild-type had default parameter values, over-expression a 50% increase in CDKA:CYCD synthesis rate and under-expression a 20% decrease. **A** Size at G1/S. **B** Duration of G1 phase. **C** Size at division. **D** Duration of the entire cell cycle.

**Fig 13:**
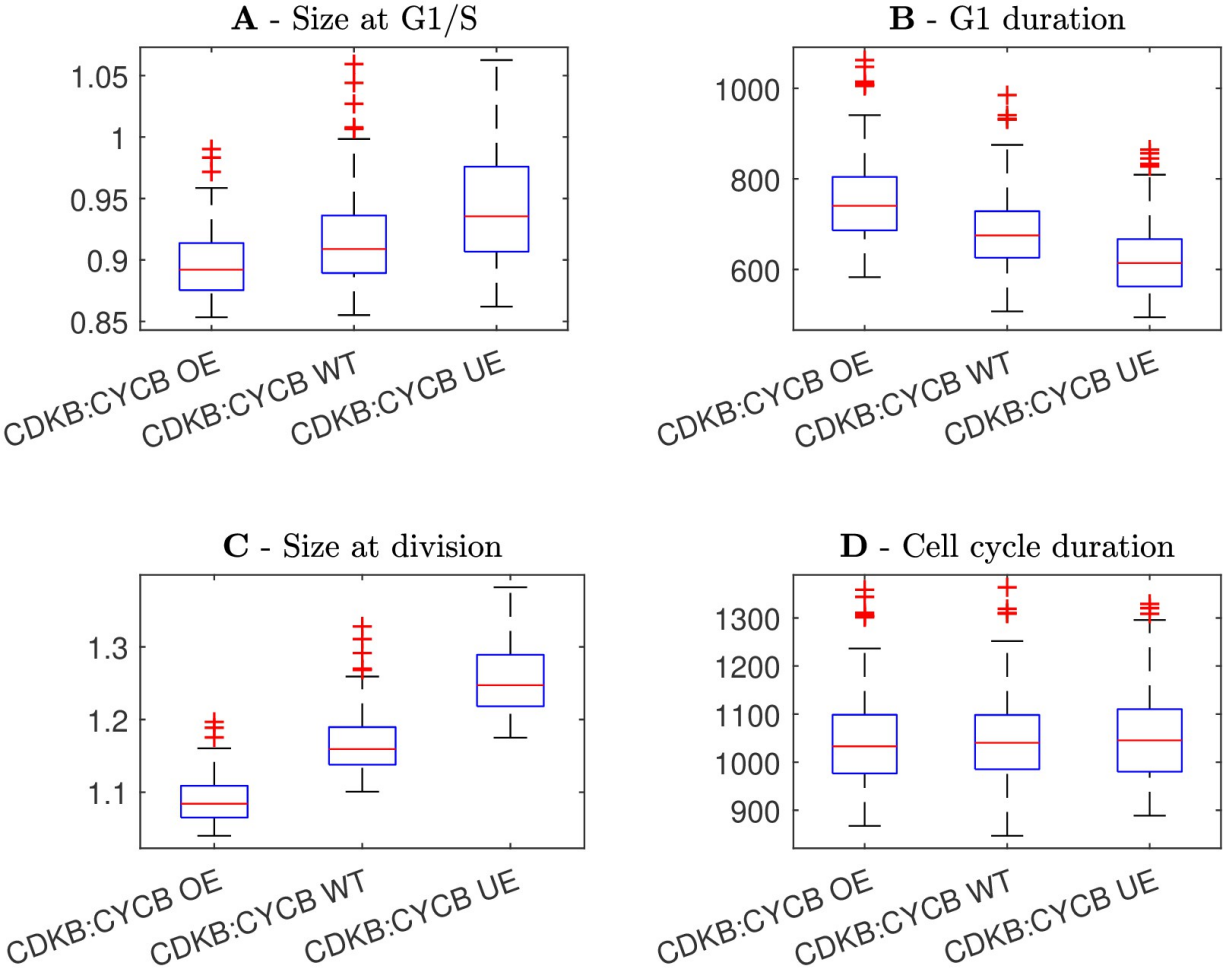
Box plots showing the distribution of cell sizes and phase durations under the assumption of CDKA/B:CYCB over-expression (OE), wild-type expression (WT), and under-expression (UE). Wild-type had default parameter values, over-expression a 50% increase in CDKA/B:CYCB synthesis rate and under-expression a 20% decrease. **A** Size at G1/S. **B** Duration of G1 phase. **C** Size at division. **D** Duration of the entire cell cycle.

The model predicts that a reduction in CDKA:CYCD expression produces overall larger cells, with greater sizes at G1/S and division (Fig 12). Similarly, a reduction in CDKA/B:CYCB expression also produces larger cells at division (Fig 13), although the effect at G1/S is much smaller, which is consistent with CDKA/B:CYCB acting in G2-phase. These simulations are in agreement with the experimental results of Jones *et al.*, who found that reductions in Cyclin D and CDKB expression led to larger cell phenotypes [13]. The predicted increase in cell size is intuitive, as Cyclin D and CDKB are cell-cycle activators. At a lower expression rate, it will take longer for the activator concentration to reach the necessary threshold, giving more time for cell growth. Perhaps less intuitively, we find that over/under-expression does not change cell-cycle duration for either CDKA:CYCD or CDKA/B:CYCB. This result agrees with Jones *et al.* who found that modification of Cyclin D and CDKB expression did not change cell-cycle duration [13].

Increased CDKA:CYCD expression caused a slight decrease in G1-phase duration, while increased CDKA/B:CYCB expression caused a slight increase in G1-phase duration, despite the fact that total cell-cycle duration was unchanged in all cases (Figs 12 and 13). This suggests that the mechanisms controlling G1/S and G2/M may compensate for each other *i.e.* longer G1 gives shorter G2 and vice-versa. We believe this result can be explained as follows: because cell growth is exponential and the activators, CDKA:CYCD and CDKA/B:CYCB, are size-dependent, smaller cells grow and express activator proteins more slowly than larger cells. This leads to a trade-off between cell size and activator expression rate which keeps cell-cycle duration approximately constant.

## 4 Discussion

Balanced growth, achieved by coupling cell division to the increase in cell volume, is crucial to cell survival as progressive changes in size over generations would eventually lead to a breakdown of biochemical processes. In this study, we developed a parsimonious model of the plant cell cycle which we used to investigate potential mechanisms of cell size control. Our modelling demonstrated that the proposed cell-cycle network exhibits limit cycles, with bistable behaviour at two checkpoints corresponding to the G1/S and G2/M transitions. Thus, the network is predicted to enable independent control of DNA synthesis and mitosis, as has been observed in plants [13].

Having considered the network in isolation, we simulated this network in growing cells to investigate possible cell-size-control mechanisms. Applying a method developed by Heldt *et al.* for budding yeast [15], we investigated whether the differential expression of activator and inhibitor proteins can explain the existence of size control at the major cell-cycle checkpoints in plants. Briefly summarising this approach, activator proteins (CDKA and Cyclin D at G1/S, and CDKA/B and Cyclin A/B at G2/M) accumulate in concentration relative to inhibitor proteins (e.g. KRP at G1/S and SMR at G2/M) until a tipping point is reached where activators dominate inhibitors, and cell-cycle progression occurs. Studies in yeast have suggested that size control can be achieved via activator proteins being synthesised in a “size-dependent” way such that they maintain roughly constant concentration (at constant rates of expression and degradation) over time, whereas inhibitor proteins are synthesised in a “size-independent” way such that the total mass of protein grows linearly over time, but the concentration gradually falls. Since activator accumulation and inhibitor dilution are coupled with cell size, the critical point at which activators dominate inhibitors necessarily occurs once the cell reaches a predictable volume.

Using numerical simulation, we show that size-independent synthesis of either KRP or RBR can explain cell-size control of the G1/S transition. The model predicts that such size-independence yields a combination of sizer- and adder-like behaviour with smaller cells (at birth) behaving more like sizers, while larger cells behaving more like adders. It is not *a priori* obvious which of these behaviours would be observed in practice. That size independence of RBR can enable size control is particularly interesting as RBR is analogous to the yeast protein Whi5, which has been shown to be an inhibitor-diluter and play a key role in controlling yeast cell size [20].

We also show that a similar mechanism operating at G2/M may control size control at mitosis independently of size control at G1/S: we find that a similar pattern of imperfect sizer-adder behaviour exists if the CDK inhibitor, SMR, is size independent. Assuming both KRP and SMR are size independent, the modelling suggests that a population of cells can maintain size homeostasis. That the cells within the population exhibit a combination of sizer and added behaviour is consistent with the recent measurements of Willis et al [3] which suggested that cells in the plant shoot apical meristem behave as ‘imperfect sizers’.

In addition to size independence, we also investigated how equal inheritance of inhibitor proteins would influence cell-cycle progression and contribute to controlling cell size. D’Ario et al [14] recently proposed that since KRP binds tightly to chromatin, KRP molecules would be equally inherited between two daughter cells, regardless of any differences in the daughter cell size. Thus, KRP concentration would be lower in a larger daughter cell, providing a mechanism for cell-cycle progression to depend on cell size. Our simulations revealed that equal inheritance of KRP contributes to cell-size control of the G1/S transition, with a combination of sizer-like behaviour for small cells and adder-like behaviour for larger cells (similar to that predicted with KRP and RBR being size independent). However, although equal inheritance of KRP is shown to contribute to size control of the G1/S transition, cell population simulations suggested that equal inheritance of KRP alone is insufficient to enable robust size control over multiple generations. The modelling suggests that combining equal inheritance of KRP with a further size-regulation of the G2/M transition would enable more robust size homeostasis within a cell population.

While there have been many cell-cycle models in other systems [5, 92, 93], there are unique features of the plant cell cycle that motivate the need for a plant-specific model [33]. In yeast, a single CDK controls both cell-cycle transitions, and there appears to be two thresholds of kinase activity for the G1/S and G2/M transitions [34, 35]. In contrast, higher eukaryotes use different CDKs at the two transitions [34]. These different CDKs may have particular importance in plants as empirical research has suggested that plant cells exhibit size control at both G1/S and G2/M [13]. Cell size is more straightforward to monitor through the cell cycle than in animal cells due to the constraining nature of the plant cell wall, and whether similar mechanisms might also operate in animals is unknown. However we also note important differences in the plant cell cycle, including the mitosis-specific expression of a novel class of CDKs (CDKB) [34, 65], and the frequent occurrence of endocycles (genome duplication without cell division) as a routine mechanism in the differentiation of cells in most plants [94].

Our model provides mechanistic understanding of the roles of the two classes of CDKs, featuring subnetworks that are shown to create biological switches at each transition and that are connected via regulation of SCF and APC. While the individual biological switches are similar to models of the G1/S transition for other organisms, the model suggests that the full plant cell cycle enables robust control of the cell size distribution. More specifically, the modelling suggests that cell-size control at both G1/S and G2/M is necessary for cell-size homeostasis over multiple generations (consistent with the experimental observations in [13]). The model predicts that size independence of RBR or KRP, for example, enables smaller cells to delay the G1/S transition; however, larger cells all transition at similar times, requiring further regulation at G2/M to mediate size homeostasis. Thus, while the modelling suggests that a range of mechanisms can contribute to size control, combining several mechanisms, with checkpoints at both G1/S and G2/M, may provide more robust size control.

In contrast to previous models of cell-size control in plants that rely on the presence of phenomenological thresholds [13], our study demonstrates how size control can be the output of the cell-cycle network dynamics, providing mechanistic insights into the roles of different cell-cycle proteins and their interactions. Our modelling thus demonstrates that the network, and its components, require certain properties in order to achieve size control, providing insights into the necessary constraints which may guide future experimental studies. The model predicts the dynamics of key proteins through the cell cycle; while these predictions are consistent with previous observations for some components, more detailed experimental imaging of further components would help verify these predictions and could lead to further iterations of both the model and our understanding. Furthermore, since the cell-cycle transitions are shown to be emergent properties of the network dynamics, the model provides a platform for investigating how these transitions are influenced by regulation of the cell cycle. It is well established that plant hormones and environmental signals influence cell-cycle proteins [95]; thus, the model could contribute to understanding how cell size is affected by both internal and external signals.

In summary, there are a number of factors which distinguish plants from other commonly studied organisms, such as yeast, bacteria, or animals; namely, the existence of plant-specific proteins (such as CDKB), or the existence of cell size checkpoints at both the G1/S and G2/M transitions. For these reasons, we surmise that insights from studies of other organisms cannot necessarily be carried over to plants, and that the plant cell cycle must be considered as a special case. Our study provides a mechanistic model of cell-cycle regulation in plant cells that readily shows size homeostasis. The developed model will provide a basis for future studies that consider how internal factors, such as plant hormones, and external environmental conditions affect plant development via cell-cycle regulation.

## 5 Methods

In this section, we provide technical details of the mathematical model of the plant cell cycle. We begin in section 5.1 with a model of protein synthesis that can account for the production of both size-dependent and size-independent proteins. In section 5.2, we provide a handful simple mathematical expressions for protein interactions under quasi-steady conditions; namely, reversible binding, phospohrylation, transcriptional control, and degradation. In section 5.3, we pose the complete cell cycle model. In section 5.4, we describe simulations of a submodel to investigate the interations between MYB3R3 and CDKA/B:CYCB. In section 5.5, we describe the parameter values and provide simulations to verify that the model and conclusions are robust to the choice of parameter values.

### 5.1 A simple model of size-dependent and size-independent protein expression

Cell size regulation may depend upon the interplay between size-dependent and size-independent proteins, but it must be explained how the difference between these two types of protein is established in the first place. One model has been proposed by Heldt *et al.* [15] in which protein transcription (hence translation) requires an interaction between RNA polymerase and a corresponding gene [15].

Consider a protein *i* is encoded by gene *i*. RNA polymerase associates reversibly with chromatin to form a complex. Let R be the mass of available RNA polymerase, *P*_*i*_ be the mass of protein *i*, *G*_*i*_ be the mass of genetic material, *C*_*i*_ be the mass of the RNA polymerase-gene complex, and *V* be cell volume. Via the law of mass action we have,

$$\begin{array}{c} {\dot{C}}_{i}=\frac{k_{i}^{+}RG_{i}}{V}-k_{i}^{-}C_{i}, \end{array}$$ (1)

along with *G*_*i*_ + *C*_*i*_ = *G*_*it*_, which is a statement of conservation of mass.

The rate of synthesis, or translation, of a protein is assumed to be proportional to the mass of encoding gene complexes, such that

$$\begin{array}{c} {\dot{P}}_{i}=k_{i}C_{i}-d_{i}P_{i}, \end{array}$$ (2)

where *d*_*i*_ is the rate of degradation.

The equation for the mass of RNA polymerase, *R*, is

$$\begin{array}{c} \dot{R}=s_{R}+\sum_{i=1}^{N_{G}}(k_{i}^{-}C_{i}-\frac{k_{i}^{+}RG_{i}}{V}), \end{array}$$ (3)

where *N*_*G*_ is the total number of genes, and *s*_*R*_ is the rate of synthesis of RNA polymerase, which is yet to be determined.

Heldt *et al.* [15] have simplified the above equations by dividing all proteins into two categories: size dependent and size-independent. For all size-dependent genes it is assumed that $k_{i}^{+}=k_{D}^{+}$ and $k_{i}^{-}=k_{D}^{-}$, and likewise $k_{i}^{+}=k_{I}^{+}$ and $k_{i}^{-}=k_{I}^{-}$ for all size-independent genes. Summing (3) for all size-dependent and size-independent genes gives,

$$\begin{array}{c} {\dot{C}}_{D}=\frac{k_{D}^{+}RG_{D}}{V}-k_{D}^{-}C_{D}, \end{array}$$ (4)

and,

$$\begin{array}{c} {\dot{C}}_{I}=\frac{k_{I}^{+}RG_{I}}{V}-k_{I}^{-}C_{I}, \end{array}$$ (5)

where *C*_*D*_ and *C*_*I*_ are the total masses of size-dependent (respectively, size-independent) gene complexes. Moreover, *G*_*D*_ + *C*_*D*_ = *G*_*DT*_, a constant (likewise *G*_*I*_ + *C*_*I*_ = *G*_*IT*_). Eq (3) becomes

$$\begin{array}{c} \dot{R}=s_{R}+k_{D}^{-}C_{D}-\frac{k_{D}^{+}RG_{D}}{V}+k_{I}^{-}C_{I}-\frac{k_{I}^{+}RG_{I}}{V}. \end{array}$$ (6)

The protein mass equation is then,

$$\begin{array}{c} {\dot{P}}_{i}=\{\begin{array}{c} k_{i}\frac{GCN_{i}C_{D}}{G_{DT}}-d_{i}P_{i}\;\text{if}\;P_{i}\;\text{is}\;\text{size-dependent},\\ k_{i}\frac{GCN_{i}C_{I}}{G_{IT}}-d_{i}P_{i}\;\text{if}\;P_{i}\;\text{is}\;\text{size-independent}, \end{array} \end{array}$$ (7)

where *GCN*_*i*_ is the number of copies of gene *i* (the gene copy number).

It is assumed that RNA polymerase itself is size-dependent, hence

$$\begin{array}{c} s_{R}=k_{R}\frac{GCN_{R}C_{D}}{G_{DT}}. \end{array}$$ (8)

Cell volume growth requires the synthesis of new proteins, most of which are size-dependent, hence the rate of volume growth is assumed to be proportional to *C*_*D*_,

$$\begin{array}{c} \dot{V}=k_{V}C_{D}. \end{array}$$ (9)

The distinction between size-dependent and size-independent proteins is ultimately caused by a difference in the values of the association and dissociation rates, with $k_{I}^{+},k_{I}^{+}\gg k_{D}^{+},k_{D}^{+}$. Consequently, the mass of size-independent gene complexes rapidly reaches a state of quasi-equilibrium, so that we may assume ${\dot{C}}_{I}\approx 0$, which together with (5), implies that

$$\begin{array}{c} C_{I}=\frac{R}{R+\frac{k_{I}^{-}V}{k_{I}^{+}}}=\frac{\left[R\right]}{\left[R\right]+\frac{k^{-}}{k^{+}}}, \end{array}$$ (10)

and

$$\begin{array}{c} \dot{R}=k_{R}\frac{GCN_{R}C_{D}}{G_{DT}}-{\dot{C}}_{D}. \end{array}$$ (11)

We can further simplify the model if we assume that the concentration of size-dependent gene complexes (not the mass) is quasi-steady; $[{\dot{C}}_{D}]=0$ and $[\dot{R}]=0$, hence $C_{D}=C_{D}^{*}V$ and *R* = R*V. This gives,

$$\begin{array}{c} \dot{V}=\mu V, \end{array}$$ (12)

where $\mu =C_{D}^{*}k_{V}$. The model under the current assumptions predicts exponential cell growth, which has been observed in many organisms [96]. It follows that,

$$\begin{array}{c} C_{I}=\frac{R^{*}}{R^{*}+\frac{k^{-}}{k^{+}}}, \end{array}$$ (13)

is constant. The equations governing protein mass simplify to

$$\begin{array}{c} {\dot{P}}_{i}=\{\begin{array}{c} \bar{k_{i}}V-d_{i}P_{i}\;\text{if}\;P_{i}\;\text{is}\;\text{size-dependent},\\ {\bar{k}}_{i}-d_{i}P_{i}\;\text{if}\;P_{i}\;\text{is}\;\text{size-independent}, \end{array} \end{array}$$ (14)

The above implies that size-dependent proteins accumulate exponentially, in proportion to cell volume, while size-independent proteins accumulate linearly over time.

### 5.2 Mathematical expressions of protein interactions

Before presenting the full model, it is useful to derive a set of simple mathematical formulae, which are used to approximate the following protein interactions:

1. Reversible binding of two proteins
2. Phosphylation of one protein by another
3. Transcriptional regulation of one protein by another
4. Degradation of one protein mediated by another

**Reversible binding.** Suppose that proteins, A and B, associate to form an unstable complex, C,

$$\text{A}+\text{B}\overset{k^{+}}{\underset{k^{-}}{⇌}}\text{C}.$$

We assume that the mass of the complex is in quasi-equilibrium, so that

$$\begin{array}{c} \dot{C}=\frac{k^{+}AB}{V}-k^{-}C=0, \end{array}$$ (15)

where *A*, *B* and *C* are the masses of A, B and C respectively, and V is cell volume. By conservation of mass, we also have *A* + *C* = *A*_*T*_ and *B* + *C* = *B*_*T*_. Assuming quasi-steady state yields

$$\begin{array}{c} C=f\left(A_{T},B_{T},V;k_{D}\right)=\frac{A_{T}+B_{T}+k_{D}V-\sqrt{{\left(A_{T}+B_{T}+k_{D}V\right)}^{2}-4A_{T}B_{T}}}{2}, \end{array}$$ (16)

where *k*_*D*_ = *k*^−^/*k*^+^.

**Phosphorylation.** Suppose that protein B is phosphorylated by A

$$\text{A}+\text{B}\overset{k_{p}^{+}}{\underset{k_{p}^{-}}{⇌}}\text{A}+{\text{B}}_{p}$$

The mass of phosphorylated B, *B*_*p*_ follows,

$$\begin{array}{c} {\dot{B}}_{p}=\frac{k_{p}^{+}AB}{V}-k_{p}^{-}B_{p}=0, \end{array}$$ (17)

where by conservation of mass *B* + *B*_*p*_ = *B*_*T*_. Under a quasi-steady state hypothesis, the mass of phosphorylated B is then

$$\begin{array}{c} B_{p}=\frac{A}{A+k_{dp}V}B_{T}=\frac{\left[A\right]}{\left[A\right]+k_{dp}}B_{T} \end{array}$$ (18)

$k_{dp}=k_{p}^{+}/k_{p}^{-}$.

**Transcriptional regulation.** Suppose that protein B is encoded by a gene, G. Transcription factor A associates with the promotor according to

$$\text{A}+\text{G}\overset{k_{t}^{+}}{\underset{k_{t}^{-}}{⇌}}\text{C},$$

where G is the mass of genetic material with which A is unassociated, and C is the mass of genetic material with which A is associated. We assume that association of A with the gene promotor induces transcription, so that the rate of synthesis of B is proportional to C,

$$\text{C}\overset{k_{B}}{\to }B,$$

We assume that the mass, C, is quasi-steady so that

$$\begin{array}{c} \dot{C}=\frac{k_{t}^{+}AG}{V}-k_{t}^{-}C=0, \end{array}$$ (19)

where *G* + *C* = *G*_*T*_, a constant. The synthesis rate of B is

$$\begin{array}{c} s_{B}=k_{B}C1_{B}\left(V\right). \end{array}$$ (20)

Then *s*_*B*_ is given by

$$\begin{array}{c} s_{B}=k_{B}\frac{A}{A+k_{t}^{-}V/k_{t}^{+}}G_{T}1_{B}\left(V\right)=r_{B}\frac{\left[A\right]}{k_{at}\left[A\right]+1}1_{B}\left(V\right). \end{array}$$ (21)

where $r_{B}=k_{B}k_{t}^{+}G_{T}/k_{t}^{-}$ and $k_{at}=k_{t}^{+}/k_{t}^{-}$. The indicator function, 1_*B*_(*V*), determines whether the gene is size-(in)dependent, and is defined as

$$\begin{array}{c} 1_{B}\left(V\right)=\{\begin{array}{c} V\;\text{if}\;\text{B}\;\text{is}\;\text{size-dependent},\\ 1\;\text{if}\;\text{B}\;\text{is}\;\text{size-independent}. \end{array} \end{array}$$ (22)

**Degradation.** The case where protein A mediates the degradation of protein B is the most straightforward,

$$\text{A}+\text{B}\overset{d}{\to }\text{A}.$$

The degradation rate, *d*_*B*_ is simply

$$\begin{array}{c} d_{B}=\frac{dAB}{V}. \end{array}$$ (23)

### 5.3 The cell cycle model

We now posit an ordinary differential equation model of the protein network outlined in section 2.1 using the principles outlined in appendices 5.1 and 5.2.

It may be simpler to conceive of the model as being separated into two ‘modules’. The G1/S module involves every protein that directly controls the initiation of DNA synthesis (hence the entry into S-phase). The G2/M module involves every protein that directly controls cell division (hence M-phase). Both of these modules form bi-stable switches due to the mutual antagonism between CDK and CDK inhibitors; CDKA versus RBR and KRP at G1/S, and CDKB versus SMR and MYB3R3 at G2/M (see sections 3.2 and 3.3 for details of the bistability at G1/S and G2/M respectively).

These modules are then linked via the ubiquitin ligase complexes SCF and APC. These proteins rapidly degrade cyclins (CYCD in the case of SCF and CYCB in the case of APC). This degradation results in a state of low CDK-cyclin concentration, effectively resetting the cell to the beginning of the cell cycle (G1 phase).

The G1/S equations can be thought of as the G1/S regulation model in isolation (likewise for G2/M). The full model is simply the combination of all equations. Note that, where multiple forms of protein A exist, we use *A*_*T*_ to refer to the total mass of protein, regardless of its binding interactions or phosphorylation. Similarly, *A*_*p*_ is the mass of phosphorylated A.

Cell growth itself is assumed to be exponential such that,

$$\begin{array}{c} \frac{d}{dt}V=r_{gr}V, \end{array}$$ (24)

where *r*_*gr*_ is the relative growth rate. As volume itself is a variable, the remaining equations are given in terms of protein mass, not concentration (as is more typical). Additionally, note that most proteins are size-dependent, hence the synthesis rate is dependent on volume. The exceptions are: RBR, KRP, MYB3R3 and SMR. We allow for the possibility that these CDK inhibitors may be size-independent.

#### 5.3.1 G1/S module

For the G1/S module, we have the following differential equations. CDKA:CYCD complexes:

$$\begin{array}{c} \frac{d}{dt}\text{CDKA}\;:\;{\text{CYCD}}_{T}=r_{ca}V-(d_{ca}+d_{ca}^{scf}\frac{\text{SCF}}{V})\;\text{CDKA}\;:\;\text{CYCD}. \end{array}$$ (25)

Note that, as a simplification, we do not track CDK and cyclin individually, as for our purposes they always act together. KRP:

$$\begin{array}{c} \frac{d}{dt}{\text{KRP}}_{T}=(r_{krp}+r_{krp}^{myb4}\frac{\text{MYB}3R4_{p}}{k_{at}^{myb4}\text{MYB}3R4_{p}+V})\;1_{krp}\left(V\right)-(d_{krp}+d_{krp}^{fbl17}\frac{\text{FBL}17}{V})\;{\text{KRP}}_{T}. \end{array}$$ (26)

E2FA:

$$\begin{array}{c} \frac{d}{dt}E2{\text{FA}}_{T}=r_{e2fa}V-d_{e2fa}E2{\text{FA}}_{T}. \end{array}$$ (27)

E2FB:

$$\begin{array}{c} \frac{d}{dt}E2\text{FB}=(r_{e2fb}+r_{e2fb}^{e2fa}\frac{E2\text{FA}}{k_{at}^{e2fa}E2\text{FA}+V})\;V-d_{e2fb}E2\text{FB}. \end{array}$$ (28)

RBR:

$$\begin{array}{c} \frac{d}{dt}{\text{RBR}}_{T}=r_{rbr}1_{rbr}\left(V\right)-d_{rbr}{\text{RBR}}_{T}. \end{array}$$ (29)

FBL17:

$$\begin{array}{c} \frac{d}{dt}\text{FBL}17=(r_{fbl17}+r_{fbl17}^{e2fa}\frac{E2\text{FA}}{k_{at}^{e2fa}E2\text{FA}+V})\;V-d_{fbl17}\text{FBL}17. \end{array}$$ (30)

We additionally have the following equilibrium equations. CDKA-CYCD-KRP complexes:

$$\begin{array}{c} \text{CDKA}\;:\;\text{CYCD}\;:\;\text{KRP}=f(\text{CDKA}\;:\;{\text{CYCD}}_{T},{\text{KRP}}_{T},V;k_{D}^{ca\;:\;krp}). \end{array}$$ (31)

Free CDKA-CYCD:

$$\begin{array}{c} \text{CDKA}\;:\;\text{CYCD}=\text{CDKA}\;:\;{\text{CYCD}}_{T}-\text{CDKA}\;:\;\text{CYCD}\;:\;\text{KRP} \end{array}$$ (32)

Phosphorylated RBR:

$$\begin{array}{c} {\text{RBR}}_{p}=\frac{\text{CDKA}\;:\;\text{CYCD}}{\text{CDKA}\;:\;\text{CYCD}+k_{dp}^{ca}V}{\text{RBR}}_{T}. \end{array}$$ (33)

Unphosphorylated RBR:

$$\begin{array}{c} \text{RBR}={\text{RBR}}_{T}-{\text{RBR}}_{p}. \end{array}$$ (34)

E2FA-RBR complexes:

$$\begin{array}{c} E2\text{FA}\;:\;\text{RBR}=f(E2{\text{FA}}_{T},\text{RBR},V;k_{D}^{e2fa:rbr}), \end{array}$$ (35)

Free E2FA

$$\begin{array}{c} E2\text{FA}=E2{\text{FA}}_{T}-E2\text{FA}\;:\;\text{RBR}. \end{array}$$ (36)

#### 5.3.2 G2/M module

For the G2/M module, we have the following differential equations. CDKB-CYCB complexes:

$$\begin{array}{c} \frac{d}{dt}\text{CDKA}/B\;:\;{\text{CYCB}}_{T}=\frac{r_{cb}+r_{cb}^{e2fb}\frac{E2\text{FB}}{k_{at}^{e2fb}E2\text{FB}+V}}{V+k_{at}^{myb3}\text{MYB}3R3}V-(d_{cb}+d_{cb}^{apc}\frac{\text{APC}}{V})\text{CDKA}/B\;:\;\text{CYCB}. \end{array}$$ (37)

Total MYB3R3:

$$\begin{array}{c} \frac{d}{dt}\text{MYB}3R3_{T}=r_{myb3}1_{myb3}\left(V\right)-d_{myb3}\text{MYB}3R3_{T}. \end{array}$$ (38)

Total MYB3R4:

$$\begin{array}{c} \frac{d}{dt}\text{MYB}3R4_{T}=(r_{myb4}+r_{myb4}^{myb4}\frac{\text{MYB}3R4_{p}}{k_{at}^{myb4}\text{MYB}3R4_{p}+V})\;V-d_{myb4}\text{MYB}3R4_{T}. \end{array}$$ (39)

Total SMR:

$$\begin{array}{c} \frac{d}{dt}{\text{SMR}}_{T}=r_{smr}1_{smr}\left(V\right)-d_{smr}\text{SMR}-d_{smr}^{cdkb}{\text{SMR}}_{p}. \end{array}$$ (40)

CDKB-CYCB-SMR complexes:

$$\begin{array}{c} \text{CDKA}/B\;:\;\text{CYCB}\;:\;\text{SMR}=f(\text{CDKA}/B\;:\;{\text{CYCB}}_{T},{\text{SMR}}_{T},V;k_{D}^{cb:smr}), \end{array}$$ (41)

Unbound CDKB-CYCB:

$$\begin{array}{c} \text{CDKA}/B\;:\;\text{CYCB}=\text{CDKA}/B\;:\;{\text{CYCB}}_{T}-\text{CDKA}/B\;:\;\text{CYCB}\;:\;\text{SMR}. \end{array}$$ (42)

Phosphorylated MYB3R3:

$$\begin{array}{c} \text{MYB}3R3_{p}=\frac{\text{CDKA}/B\;:\;\text{CYCB}}{\text{CDKA}/B\;:\;\text{CYCB}+k_{dp}^{cb}V}\text{MYB}3R3_{T}, \end{array}$$ (43)

Unphosphorylated MYB3R3:

$$\begin{array}{c} \text{MYB}3R3=\text{MYB}3R3_{T}-\text{MYB}3R3_{p}. \end{array}$$ (44)

Phosphorylated MYB3R4:

$$\begin{array}{c} \text{MYB}3R4_{p}=\frac{\text{CDKA}/B\;:\;\text{CYCB}}{\text{CDKA}/B\;:\;\text{CYCB}+k_{dp}^{cb1}V}\text{MYB}3R4_{T}. \end{array}$$ (45)

Phosphorylated SMR:

$$\begin{array}{c} {\text{SMR}}_{p}=\frac{\text{CDKA}/B\;:\;\text{CYCB}}{\text{CDKA}/B\;:\;\text{CYCB}+k_{dp}^{cb2}V}{\text{SMR}}_{T}. \end{array}$$ (46)

Unphosphorylated SMR:

$$\begin{array}{c} \text{SMR}={\text{SMR}}_{T}-{\text{SMR}}_{p} \end{array}$$ (47)

#### 5.3.3 ‘Linking’ molecules (ubiquitin ligases)

SCF:

$$\begin{array}{c} \frac{d}{dt}\text{SCF}=(r_{scf}+r_{scf}^{e2fb}\frac{E2\text{FB}}{k_{at}^{e2fb}E2\text{FB}+V})\;V-d_{scf}\text{SCF}. \end{array}$$ (48)

APC:

$$\begin{array}{c} \frac{d}{dt}\text{APC}=(r_{apc}+r_{apc}^{myb4}\frac{\text{MYB}3R4_{p}}{k_{at}^{myb4}\text{MYB}3R4_{p}+V})\;V-d_{apc}\text{APC}. \end{array}$$ (49)

### 5.4 Simulations of interactions between CDKA/B:CYCB and MYB3R3

**Fig 14:**
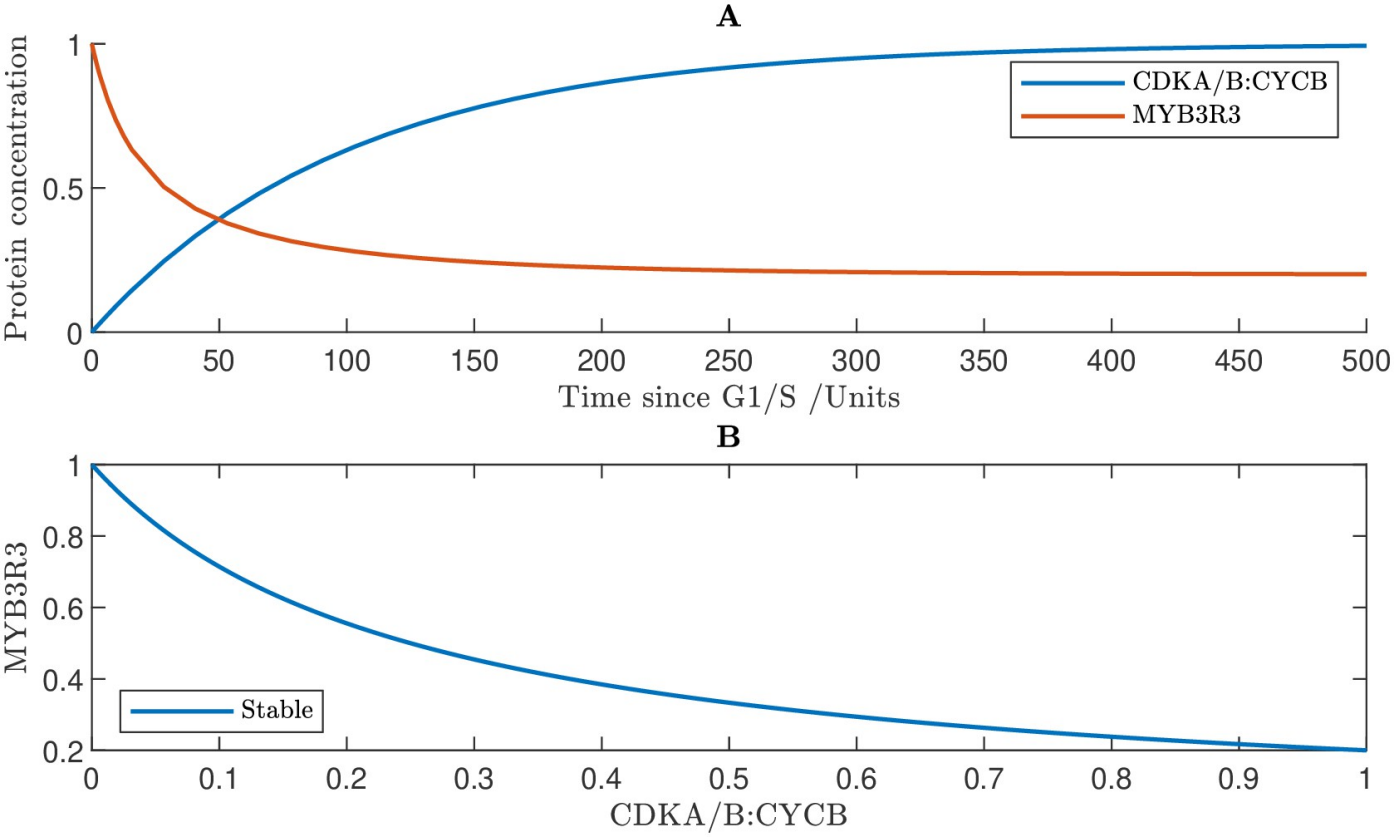
Modelling the interaction between CDKA/B:CYCB and MYB3R3. **A** Simulated time course showing the concentrations of CDKB and un-phosphorylated MYB3R3. **B** Bifurcation diagram showing MYB3R3 steady states. Note that this system exhibits no hysteresis, in contrast with the results in Fig 5 for a network that includes SMR. Although there is mutual inhibition between MYB3R3 and CDKB, this is not sufficient to establish a bistable switch. In this model, MYB3R3 may control entry into G2/M, but only by modulating expression of CDKB.

To understand the relationship between CDKA/B:CYCB and MYB3R3, we simulated a submodel in which SMR synthesis is prohibited (*r*_*smr*_ = 0), so that at the G2/M transition, we are left with interactions between CDKA/B:CYCB and MYB3R3. Results, presented in Fig 14B, show that this restricted system is mono-stable, so that the system does not ‘switch’ between states, but rather MYB3R3 activity gradually decreases as CDKA/B:CYCB activity increases (Fig 14A). It is intuitively clear that mutual inhibition between two (or more) proteins may give rise to a bi-stable switch, but this is not guaranteed. CDKA/B:CYCB and MYB3R3 are mutually inhibiting, but the resulting network is mono-stable. We therefore expect that interactions between CDKs and MYB transcription factors alone are insufficient, and that other proteins (such as SMR) are required to establish a biological switch regulating the G2/M transition.

### 5.5 Parameter values

Simulations are performed using the parameter values given in Table 2. For most parameters we choose values used in previous modelling studies. To select an appropriate value for the cell growth rate, we used the cell-cycle duration predicted from the full cell cycle network model (Fig 3), and chose the growth rate so that the cells doubled in size during this time period.

**Table 2:** Table of default parameter values for each protein. Parameters take these values in model simulations unless otherwise stated. Parameter values marked with an asterisk (*) are reproduced from [37]. As far as we are aware, suggestions for the other parameter values are not currently available in the literature.

Protein	Parameters
CDKA:CYCD	$r_{ca}=0.01^{*},d_{ca}=0.01^{*},d_{ca}^{scf}=0.0,d_{ca}^{apc}=0.5,k_{D}^{ca:krp}=0.01^{*}$
KRP	$r_{krp}=0.01^{*},r_{krp}^{myb4}=0.01,d_{krp}=0.01^{*},d_{krp}^{fbl17}=1^{*}$
E2FA	$r_{e2fa}=0.01,d_{e2fa}=0.01,k_{D}^{e2fa:rbr}=0.001^{*}$
E2FB	$r_{e2fb}=0,r_{e2fb}^{e2fa}=0.01,d_{e2fb}=0.01$
RBR	$r_{rbr}=0.01,d_{rbr}=0.01,k_{dp}^{ca}=0.25^{*}$
FBL17	$r_{fbl17}=0^{*},r_{fbl17}^{e2fa}=0.1^{*},d_{fbl17}=0.1^{*}$
CDKA/B:CYCB	$r_{cb}=0,r_{cb}^{e2fb}=0.3,k_{I}^{myb3}=10,k_{at}^{e2fb}=0.001,d_{cb}=0.01,d_{cb}^{apc}=0.1,k_{D}^{cb:smr}=0.001$
MYB3R3	$r_{myb3}=0.01,d_{myb3}=0.01,k_{dp}^{cb}=0.125$
MYB3R4	$r_{myb4}=0.01,r_{myb4}^{myb4}=0.11,k_{at}^{myb4}=,d_{myb4}=0.1,k_{dp}^{cb2}=0.125$
SMR	$r_{smr}=0.01,d_{smr}=0.01,d_{smr}^{cdkb}=0.1,k_{dp}^{cb2}=0.25$
SCF	$r_{scf}=0,r_{scf}^{e2fb}=0.01,k_{at}^{e2fb}=0.001,d_{scf}=0.01$
APC	$r_{apc}=0,r_{apc}^{myb4}=0.01,k_{at}^{myb4}=0.001,d_{apc}=0.01$

**Fig 15:**
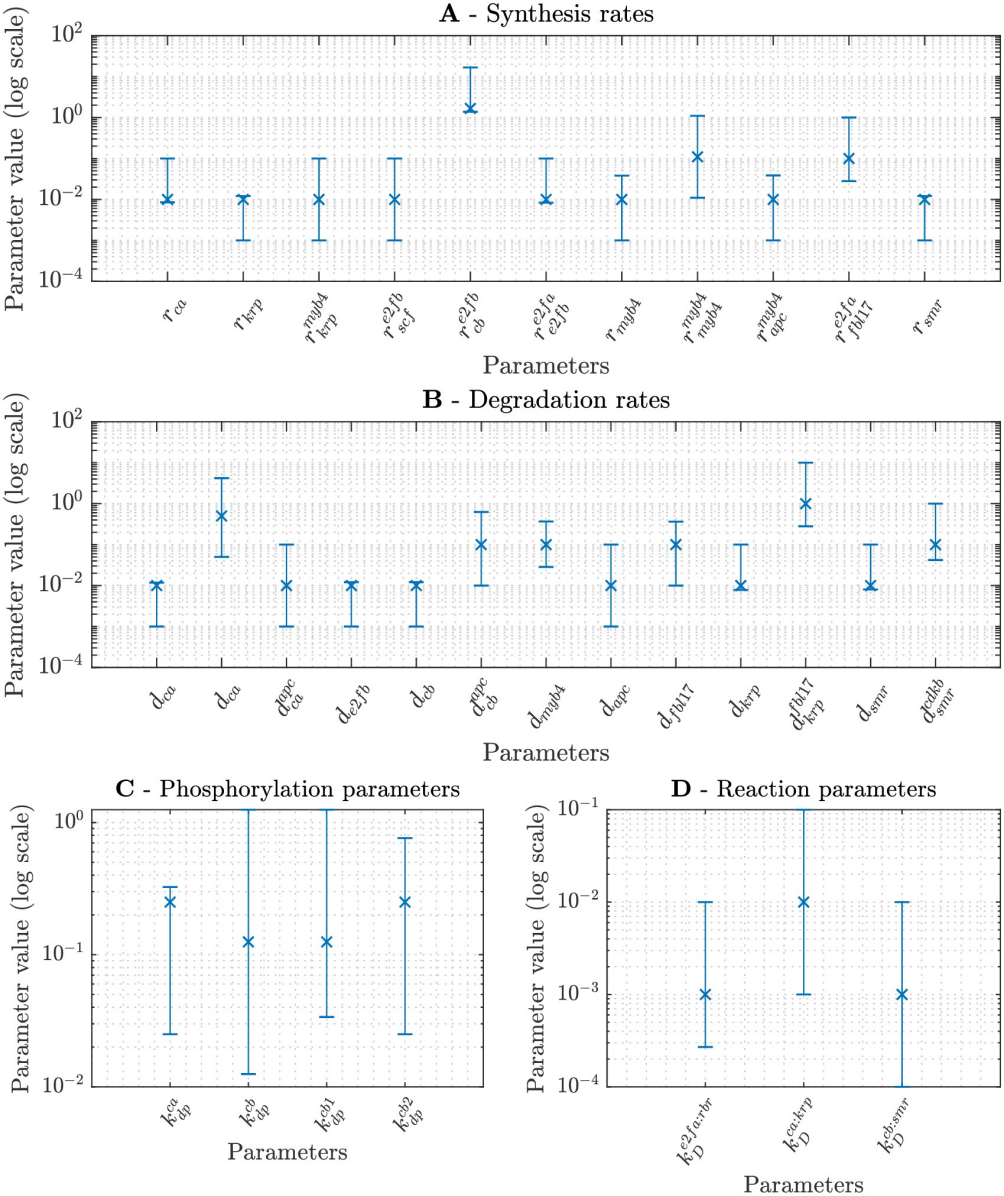
Investigating the robustness of limit cycle solutions to parameter variation. Each parameter was increased and decreased, one at a time, by at most a factor of 10 in either direction. The range of values in which limit cycle solutions were observed is indicated by bars. Default parameter values (as given in Table 2) are marked with an x.

To ensure the cell-cycle network model is robust to the choices of parameter values, we varied each parameter and tested whether the model produced limit-cycle solutions. We found that the model produces limit cycles for a range of each parameter value, although it is particularly sensitive to certain parameter values (Fig 15). For example, these results show that the CDKA:CYCD synthesis rate (*r*_*ca*_) cannot be too small and the KRP synthesis rate, *r*_*krp*_ cannot be too large, as the concentration of CDKA:CYCD needs to reach a sufficient level to overpower the inhibition via KRP and mediate the G1/S transition.

We also performed simulations to verify that the parameter choices do not affect our conclusions that size homeostasis is achieved via size control at both G1/S and G2/M. For each parameter, we considered a lower and higher value (using on the lower and upper bounds for which limit cycles were produced) and then simulated the multicell model assuming that KRP and SMR are size independent. Calculating the dispersion coefficients in each case (as in Table 1) showed that size control at both transitions enabled size homeostasis over multiple generations (see S1 Table).

## Supporting information

S1 Table Evaluation of the robustness of the cell size control to the parameter values. (PDF)
