# Supplementary material for: Modelling how plant cell-cycle progression leads to cell size regulation
Source: PLoS Comput Biol. 2023 Oct 20;19(10):e1011503. doi: 10.1371/journal.pcbi.1011503 (PMC10653611; doi:10.1371/journal.pcbi.1011503)
Supplement: S1 Table — (PDF) [file pcbi.1011503.s001.pdf]

Modelling how plant cell-cycle progression leads to cell size regulation
Supplementary Material

D. Williamson, W. Tasker-Brown, J. A. H. Murray, A. R. Jones, L. R. Band

	Lower			Upper		
Parameter	Birth	G1/S	Division	Birth	G1/S	Division
r_{ca}	0.071	0.028	0.024	0.072	0.063	0.013
r_{krp}	0.094	0.068	0.035	0.093	0.035	0.031
r_{krp}^{myb4}	0.089	0.037	0.032	0.082	0.034	0.029
r_{scf}^{e2fb}	0.087	0.036	0.031	0.074	0.031	0.026
r_{cb}^{e2fb}	0.081	0.036	0.029	0.079	0.023	0.023
r_{e2fb}^{e2fa}	0.097	0.042	0.035	0.101	0.03	0.03
r_{myb4}	0.103	0.042	0.036	0.096	0.04	0.034
r_{myb4}^{myb4}	0.077	0.03	0.026	0.095	0.061	0.054
r_{apc}^{myb4}	0.084	0.034	0.03	0.077	0.034	0.029
r_{fbl17}^{e2fa}	0.09	0.036	0.032	0.071	0.049	0.027
r_{smr}	0.069	0.019	0.018	0.065	0.03	0.025
d_{ca}	0.094	0.064	0.049	0.07	0.026	0.023
d_{ca}	0.08	0.027	0.023	0.089	0.053	0.047
d_{ca}^{apc}	0.071	0.03	0.026	0.067	0.028	0.024
d_{e2fb}	0.084	0.034	0.031	0.098	0.041	0.033
d_{cb}	0.098	0.035	0.033	0.074	0.033	0.027
d_{cb}^{apc}	0.075	0.037	0.032	0.078	0.021	0.017
d_{myb4}	0.064	0.03	0.025	0.095	0.038	0.034
d_{apc}	0.11	0.072	0.067	0.082	0.005	0.004
d_{fbl17}	0.064	0.03	0.025	0.094	0.036	0.032
d_{krp}	0.087	0.033	0.029	0.078	0.071	0.022
d_{krp}^{fbl17}	0.084	0.033	0.029	0.076	0.052	0.029
d_{smr}	0.08	0.037	0.03	0.093	0.015	0.015
d_{smr}^{cdkb}	0.088	0.036	0.03	0.091	0.033	0.031
k_{dp}^{ca}	0.082	0.032	0.028	0.073	0.032	0.027
k_{dp}^{cb}	0.083	0.035	0.03	0.065	0.027	0.023
k_{dp}^{cb1}	0.072	0.031	0.027	0.093	0.031	0.026
k_{dp}^{cb2}	0.097	0.037	0.033	0.065	0.029	0.023
$k_{D}^{e2fa:rbr}$	0.068	0.027	0.024	0.085	0.058	0.032
$k_{D}^{ca:krp}$	0.063	0.025	0.022	0.083	0.047	0.035
$k_{D}^{cb:smr}$	0.087	0.038	0.032	0.085	0.026	0.024

Table S1: Investigating the robustness of cell size control to parameter variation. Coefficients of dispersion for a population of cells at birth, G1/S and division. Values are computed for lower and upper parameter values as indicated. The lower and upper values are chosen to be the minimum and maximum values for which the model produces a limit-cycle solution (as shown in figure 15 of the main text). It is assumed that KRP and SMR are size-independent.
