# Supplementary material for: Modelling how plant cell-cycle progression leads to cell size regulation
Source: PLoS Comput Biol. 2023 Oct 20;19(10):e1011503. doi: 10.1371/journal.pcbi.1011503 (PMC10653611; doi:10.1371/journal.pcbi.1011503)
Supplement: Attachment — Submitted filename: Plant cell cycle modelling - response to reviewers-4.pdf [file pcbi.1011503.s002.pdf]

Dear Editor,

Very many thanks for considering our manuscript ‘Modelling how plant cell-cycle progression leads to cell size regulation’. We thank the reviewers for their detailed consideration of this manuscript and constructive comments. We have carefully considered each of these suggestions and addressed them in turn. As described in detail in the document below, these suggestions have led to several key improvements, including:

- Introducing additional simulations that predict how key loss-of-function mutations affect cell sizes and cell-cycle lengths (in agreement with previously published experimental measurements for these mutants),
- Incorporating a detailed parameter survey to demonstrate that our model and conclusions are robust to the parameter choices,
- Extending the introduction to include more details on previous models of size control and how the plant cell-cycle relates to that in other systems.

We hope that you find our improved manuscript suitable for publication in PloS Computational Biology.

Yours Sincerely,

Leah Band and Angharad Jones (Co-corresponding authors)

Reviewer #1: Balanced growth and division is essential for all forms of life. It is achieved by size control mechanisms operating either before DNA replication or preceding entry into mitosis. It is also important to emphasize that cells do not 'measure' their size per se, but rather they are sensing the ratio of cell volume relative to DNA content. Evidence for size control has been well-known for a long time and numerous theoretical models were formulated for explanation. These models broadly speaking fall into one of the following three categories: inhibitor-dilution, activator-accumulation and titration of nuclear sites. Recently, the first two concepts were provided with experimental verification in budding and fission yeast as well as in human cells. In contrast, study of plant cell size control has led to a novel version of titration of nuclear sites model where a cell cycle inhibitor is equally distributed between daughter cells by chromatin binding. All of these background information is provided by this manuscript. The authors develop a comprehensive cell cycle model which shows limit cycle oscillation and it contains two bistable switches operating at G1/S and G2/M transitions. The oscillatory solution of the plant cell cycle control system is impressive. In case all the cell cycle regulators are synthesized in a size-dependent manner the model does not show any size control as expected. Therefore they supplement the model with dilution of size-independent inhibitor or with chromatin mediated equal-inheritance of inhibitor. They find that only combination of two mechanisms provides perfect size control. The following points should be addressed in a revised manuscript.

Major points:

1. The authors should be aware and make it clear that dilution of size-independent inhibitor and chromatin-mediated equal distribution of an inhibitor are not sufficient to provide size control on their own, because both mechanisms require further kinetic constraints and/or assumptions about the expression mechanism. For example size-independent inhibitor-dilution could work without equal-distribution at division if the inhibitor turnovers rapidly And the rate of (amount) synthesis is proportional to the gene copy number. On the other hand, chromatin-mediated equal-inheritance of an inhibitor, as proposed by D'Ario et al., requires the degradation of the unbound fraction of the inhibitor in order to keep the number of inherited molecules proportional to the DNA content.

We have provided further details to fully explain the assumptions behind these mechanisms. Motivated by both this suggestion (and that of reviewer 2), we have rewritten the description of the size-independent inhibitor-dilution mechanism in the introduction (see p3). We now explain that this mechanism relies on further constraints on synthesis and/or turnover. We also explain that size-independence can be created by the rate of synthesis depending on gene copy number. For the description of the chromatin-mediated equal distribution mechanism, we have added text to the introduction (p4) explaining that this mechanism requires rapid degradation of the unbound inhibitor. We have also added text to section 3.7 (p22), explaining how this requirement is represented in our simulations of the equal inheritance mechanism.

2. Assuming constant rate of synthesis for size-independent inhibitor implies a rate-limiting step in synthesis, e.g. limiting the transcription rate by gene-copy number. In this case, the synthesis rate cannot constant during the whole cell cycle because of the duplication of the DNA content.

The idea that gene duplication at the G2-M transition would double the synthesis rate for size-independent genes is an interesting suggestion. However, the current consensus is that DNA replication does not affect transcription rates, as described in the review of Ben-Moshe and Itzkovitz (2016) *eLife* **5**:e14953, and the references therein. We have added text to explain and justify this assumption in the multicell model description in section 2.2 (see p9).

Minor points:

1. The trajectories on the phase plane in Fig.3A and B desperately require arrows to indicate the direction of rotation.

We have added these.

2. On page 20, it is claimed that MYB3R3 (a G2/M CDK inhibitor) cannot provide the basis for size control and conclude 'that this is because MYB3R3 is not directly involved in the hysteretic feedback loop that establishes a bistable switch at G2/M'. It is difficult to follow the authors' argument, because MYB3R3 is in a double-negative feedback with CDK. The authors should make it clearer why it doesn't contribute to the bistable switch.

We agree that this previous sentence required more details. We have rewritten this to clarify that while MYB3R3 is in a double-negative feedback with CDK, its contribution is to modulate the expression of CYCB rather than contributing directly to the bistability (see section 3.5, p18). Furthermore, to clarify this point, we have added an extra figure panel (Figure 5C), and incorporated extra simulations of a subnetwork into a new Methods section (section 5.4, p38-39). These new results show that there is a monostable relationship between MYB3R3 and CDKA/B:CYCB and thus MYB3R3 does not contribute directly to the G2/M biological switch.

3. Fig 9 shows the size homeostasis phenotype of a system with size-independent inhibitor-dilution regulated transitions at G1/S and G2/M. It is not clear why 'Birth to G1/S' has an adder phenotype, while 'G1/S to division' has the two-tier sizer/adder phenotype. This may be because the G2/M sizer ensures the birth size is large enough for the G1/S sizer to be cryptic, but the paper makes no comment about it.

We agree that these simulations suggest that the added volume during G1 is independent of cell size at birth, suggesting that birth size is sufficiently large that the cells are in the 'adder' regime shown in Figure 6. We have revised the text to make this point clearly (see section 3.6, p21).

<u>Reviewer #2:</u> The paper presents an ODE model for cell cycle and cell size homeostasis in the plant system. ‘In the plant system’ means that the variables and mechanisms at play in the ODEs are taken from the plant literature – as such they will invariably be incomplete (and unlikely to be completely correct), but the attempt is still worthwhile. The analysis is done in a clear straightforward manner. I liked the isolation of the limit cycles for G1/S and G2/M, for example. It also was striking that the equations worked without proposing any cooperativity (no Hill coefficients greater than one), which is somewhat unusual in this sort of paper and suggests a reasonably robust model. In general the model is very carefully explained, and the analysis of how it plays out through multiple cell cycles to give (or not give) cell size homeostasis is done well and explained clearly.

The model is fairly complex in terms of number of variables, and there is a commensurately substantial parameter set. The parameters are (as far as the reader can tell) unconstrained by empirical data, which is always an issue. This is because the literature might propose a general mechanism expressible by an ODE, e.g. ‘KRPs are degraded in response to CDK phosphorylation’ but the functional importance of this claim in the model will depend on the parameters, which will govern how fast this degradation occurs in contrast to baseline degradation, changes in CDK levels (KRP targets) etc. The authors can’t be faulted for not having empirical data for these parameters, but as a fallback it would be useful to try some systematic parameter variations to see how ‘breakable’ the system is. Such an exercise also has the potential to identify the least secure aspects of the model, potentially directing empirical examination of the critical parameter values.

On a related point, many of the conclusions of the paper are based on simulation of results with the current parameter set, which one supposes was optimized initially to get the ‘wild-type’ model to work. So the questions (e.g.) as to whether or to what extent a successful homeostatic model requires both the described G1/S and the G2/M controls might be answered differently with variation of parameter values.

We fully agree that it is important to verify that our conclusions are robust to our choice of parameter values. As detailed below, we have added a section to the Methods (section 5.5, p39-40 to describe the parameter values and have added detailed parameter surveys to verify that our results are robust to these parameter choices:

- Where available, parameter values were chosen based on values in a previous publication, and we have now incorporated the reference into Table 2.
- Many of the other parameters in the network model are not available and we now state this in the caption of Table 2.
- We have added text to explain that the cell growth rate was chosen by using the cell-cycle duration predicted from the full cell cycle network model (Figure 3), to calculate the growth rate for which cells doubled in size during this time period (see Methods section 5.5, see p39).

- We have added new simulations to show that the cell-cycle network model produces limit cycle solutions for a range of values for each parameter (see new Figure 15, p40). These results are discussed in section 3.1 (see p12).

- We have added new simulations showing that the parameter choices do not affect our conclusions that size homeostasis is achieved via size control at both G1/S and G2/M. We used a one-at-a-time method to vary each parameter in turn and then simulated the full cell cycle network assuming that KRP and SMR are size independent. Calculating the dispersion coefficients in each case (as in Table 1) showed that size control at both transitions enabled size homeostasis over multiple generations. These new results are provided in as a Supplementary Table S1 and discussed in section 3.6 (p21) and Methods section 5.5 (p39).

My most general question about the model really is the following: is this truly a plant-specific model, or more a generic model with plant protein names, constrained in a limited way by empirical data? After all, this is a model in the Tyson-Novak vein, of which there have been quite a few published for diverse organisms, but essentially all of which rely critically on bistable states and switches, as does the present model. I realize that the specifics in this model are derived from the plant literature and therefore do not match any of the Tyson-Novak efforts, but I do wonder how much of a translation would be required. Provided the promoters and inhibitors of G1/S and G2/M have the correct signs of activating and inhibiting each other, maybe the plant-specific features of the present model are frequently not critical. Put another way, a model will plausibly require bistable switches to give the expected sort of behavior; but the biochemical mechanisms by which the required mutual inhibition or activation is achieved could be of many different kinds. To take one example, would the current model in which APC is activated by transcription really not work if APC was instead activated by CDK phosphorylation?

This is an interesting point. There are important differences between the plant cell cycle and that of animals or yeasts/fungi, and we have added text to both the introduction (p4-5) and the discussion (p28-29) describing these differences and providing a clearer motivation for our plant-specific model.

In yeast, a single CDK controls both transitions, and there appears to be two thresholds of kinase activity required for first the G1/S transition, and then a higher level for G2/M (Coudreuse and Nurse, 2010; Nature 468:1074-1079). Higher eukaryotes in contrast exert additional control by using different CDKs at the two transitions. Furthermore, empirical research has suggested that plant cells exhibit size control at both G1/S and G2/M (Jones et al. 2017; Nat Commun. 8:15060), in contrast to most organisms which appear to regulate cell size by controlling the duration of the G1 phase alone (Xie et al. 2022; Ann Rev Cell Dev Biol. 38:291-319). Our model provides a mechanistic understanding of the roles of the two classes of CDKs, featuring subnetworks that are shown to create biological switches at each transition and that are connected via regulation of SCF and APC. While the individual biological switches are similar to

models of the G1/S transition for other organisms, the unique features of our plant-specific model suggests that the full plant cell cycle enables a robust control of the cell size distribution.

Regarding the question of the role of APC, we have answered this after the next comment, so please see this below.

To comment generally on the APC: APC almost surely promotes mitosis by other mechanisms, most prominently by degradation of securin (PATRONUS). In yeast and animal systems, APC is activated by cyclin B-CDK, and this is an important feature of control. Another feature that is very important in yeast and animal systems is alternative activation of APC by Cdh1 or by Cdc20 (the former being inhibited by cyclin-CDK, the latter activated). Both CDH1 and CDC20 are conserved in plant genomes; of course, the control circuitry around them might be quite different. So, do we take the present model as making a prediction that cyclin B/Cdc20/Cdh1 control of APC activity doesn't matter in plants? Or that these things are somehow bundled into existing variables and parameters? Or (an unsettling thought) that these things are working in plants and are important there, but the present model can still make an apparently successful simulation while ignoring them altogether?

The roles of cdc20 and cdh1 in plants are much less well understood than in other systems, and whether they contribute to plant cell size control has not been clearly established. It has been demonstrated that knocking down certain isoforms of cdc20 in Arabidopsis affects cell number, but not cell size (Kevei et al. 2011, PloS One, 6(6): e20618). Furthermore, it was recently shown that although most Arabidopsis CDC20 degrons share a high sequence similarity to other eukaryotes, there is also some significant differences at the protein level that mean that the roles may not be identical (Cosma et al. 2022, Front Physiol, 13: 938688). We further note that research into ccs52a (the plant homologue of cdh1) has mainly revealed its role during the switch from mitotic cycles to endocycles (Larson-Rabin et al. 2009, Plant Physiol, 149(2):874-84; Kasili et al. Genetics, 2010, 185(1):257-68); whether ccs52a also plays a key role in mitotic cycles has not been established.

We emphasise that our aim was to create a parsimonious model. We feel that the current literature on the roles of CDC20 and CDH1 in plants (described above) does not warrant their inclusion. We note that the current model has APC transcription promoted by CYCB via its phosphorylation of MYB3R4, and we envisage an additional mechanism for CYCB regulation of APC would likely provide additional robustness.

The paper does not make much of an effort to show how the model does (or doesn't) accord with available mutant data, nor does it go far to propose predictions (for mutants or physiological perturbations) that might be critical for testing the model. This is, again, an exercise that would distinguish the model as being 'about' real plants, as opposed to showing only that a theoretical model of this structure is potentially workable and internally consistent. Please understand, I do not minimize the effort that goes into the latter, but I think after all the Tyson-Novak school of literature, the theoretical contribution here is not adequate to stand on its own; and recently

published ODE models do try (to varying degrees) to come to terms with empirical physiological and mutant data.

This is an excellent suggestion and we have performed new simulations of the full model for two key mutants: *cycd3* and *cdkb1*. Data in Jones et al. 2017, Nat Comms. 8:15060 (in Figures 5 and 8 of this reference) showed that *cycd3* has an increased G1, reduced G2/M and larger cell size whereas *cdkb1* has reduced G1, increased G2/M and larger cell size. We were delighted to see that our model recapitulated these observations. These new results can be found in a new subsection 3.8 (p25-26) and new Figures 12 and 13.

Minor comments: Ref 15 is cited also as ref 9. The first citation of ref 15 (bottom of p. 2) is really an inadequate exposition of the Whi5 hypothesis. An essential part is that Whi5 is synthesized at a constant amount per cell cycle, independent of cell size. This idea seems to be cited here mostly from the theoretical work in ref 3 (2018), while the idea was originally proposed and supported by strong empirical results in ref 15/9 (2015).

We apologise for this error in our referencing, and have corrected this. We have revised this introduction text to ensure that the Whi5 hypothesis is more clearly explained, including the importance of the Whi5 synthesis per cell cycle being independent of cell size (see p3). We have ensured that this hypothesis is fully acknowledged to the original Schmoller et al 2015 paper.

p. 3: 'functionally conserved' is not, for me, correct language to suggest the relationship between E2F and SBF or between Rb and Whi5. 'Conservation' implies a common ancestry following which some features are retained ('conserved'). These cases in contrast are almost surely due to convergence of unrelated proteins to carry out similar network roles.

We fully agree and have rewritten these sentences to say that these processes as being functionally similar or functionally analogous (see p4).

p. 3: to summarize the budding yeast literature as showing they act as 'adders' seems like a considerable oversimplification of a lot of literature, showing a strong though imperfect sizing component to the budding yeast G1/S control. This includes the Whi5 story that has already been cited. (This literature goes back at least to the early 1980's [e.g. Work by Alan Wheals on 'sloppy size control'] and is still an area of active work. I understand this paper is not a review of the literature but this goes too far in the other direction).

Here we wished to emphasise the phenomenological differences in cell size control in different organisms. However, we agree that our original description was an oversimplification and so we have added a more detailed description of the empirically observed behaviours that have been observed in different organisms, including budding yeast, and added earlier references to the key theoretical mechanisms (see p4).

Earlier in the introduction, we have also added citations to early works in this area (see p3).

Reviewer #3: Manuscript entitled „Modelling how plant cell-cycle progression leads to cell size regulation" dealing with different cell size controlling mechanisms operating at the two major cell cycle transitions G1-S and G2-M in plants. Authors are experts on this field as they have already published excellent results focusing on cell size control functioning during plant cell cycle (eg. Jones et al., 2017; 2019). Here on the basis of mathematical models they do simulations in cell populations, and suggested integrating size-control mechanisms at both G1/S and G2/M is essential to maintain cell-size homeostasis.

Recent new results emerging from this field including the equal inheritance of KRP4 at G1-S (D'Ario et al., 2021) and the role of SMRs in the control of G2-M (Nomoto et al., 2022) indicating that both transitions in cycling cells could serve as size controller. The authors built these new cell size-mechanisms into their simulations, and resulted in an interesting model. One of their major findings is that equal inheritance of KRP, a plant specific CDK inhibitor functioning in G1 could not alone be itself a "sizer" but in combination with a G2 CDK inhibitor SMR protein. This model might explain why plant cells specifically control their size at two major cell cycle transition points.

In yeast and probably in animal cells as well, an increasing CDK activity drives unidirectional cell cycle progression to one phase to another and reaching its maximum in M-phase. This look different in plant cells as two peaks of CDK activity was detected at the major transition points (at G1/S and G2/M, respectively). KRPs and SMRs are the inhibitors of different CDKs like the types of CDKA and CDKB. Why do plant cells have these unique regulations? How do they control/fine tune each other? These are rather theoretical questions.

We thank the reviewer for their positive comments on our work. We agree that how the plant cell-cycle is related but distinct from the cell cycle in other biological systems is an interesting aspect of our work. We have now added further details to the introduction describing these differences (see p4-5), and revisit these ideas in the discussion when explaining the relevance of our results (see p28-29).

Minor points.

Figure 1. Neither dashed nor dotted lines in their model.

We apologise that these sentences in the legend were incorrectly retained from a previous draft - these have now been deleted.

Figure 3. I am not sure that experimental data supporting all these simulations: Do really the concentrations of CDKA:CYCD and CDKB:CYCB complexes peak at the same time in the G2 phase? E2FA and E2FB were also shown to have different cell cycle dependency as E2FA peaks in S while E2FB is more constitutive during cell cycle. Concentration of RBR was also shown to be unchanged throughout cell cycle but supposed to be its phosphorylation level (and consequently its activity) changes.

These results in Figure 3 are model predictions, and as far as we are aware these dynamics have not been observed in this detail for all components. However, where available the predictions do match observations, and we have added text to section 3.1 (p12) describing this available data. For example, the model accurately predicts the well-documented bursts of CYCB (Colón-Carmona et al. 1999 Plant J. 20:503-508) and MYB3R4 (Yang et al. 2021 Science, 371: 1350-1355) in the late G2 phase and G2/M transition respectively. The model also predicts G2 phase-specific expression of FBL17 observed in (D'Ario et al, 2021 Science, 372: 1176-1181).

For RBR, the model does indeed assume that total RBR levels are unchanged throughout the cell cycle. We now explain that this model assumption is based on observations that RBR transcription rates are not regulated during cell-cycle progression (Menges et al, 2005. Plant J. 41: 546-566), see new text on p7..

Further detailed experimental analysis will be required to validate all of the predictions, but the different profiles that the model predicts may help us understand better the roles of different family members - e.g., the early but sustained peak of CYCD versus the sharp peak of CYCB and the potential differences between E2FA and E2FB. We have added a sentence to the discussion suggesting that further experimental imaging could help verify these predictions on the dynamics of key proteins (see p29).

Figure 5. Why SMOS1:SCL28 mentioned in the legend? Why they are not presented in the graph? Some introductions require.

We apologise that this part of the legend was incorrectly retained from a previous draft - this has now been deleted.

On top of page 27: "Cyclin B inhibiting transcription factor, SMR, is size independent" SMR is a CDK inhibitor.

We have corrected this error.
